# Individual Risk Prediction for Sight-Threatening Retinopathy of Prematurity Using Birth Characteristics

**DOI:** 10.1001/jamaophthalmol.2019.4502

**Published:** 2019-11-07

**Authors:** Aldina Pivodic, Anna-Lena Hård, Chatarina Löfqvist, Lois E. H. Smith, Carolyn Wu, Marie-Christine Bründer, Wolf A. Lagrèze, Andreas Stahl, Gerd Holmström, Kerstin Albertsson-Wikland, Helena Johansson, Staffan Nilsson, Ann Hellström

**Affiliations:** 1 Department of Ophthalmology, Institute of Neuroscience and Physiology, Sahlgrenska Academy, University of Gothenburg, Gothenburg, Sweden; 2 Statistiska Konsultgruppen, Gothenburg, Sweden; 3 Institute of Health Care Science, Sahlgrenska Academy, University of Gothenburg, Gothenburg, Sweden; 4 Department of Ophthalmology, Boston Children’s Hospital, Harvard Medical School, Boston, Massachusetts; 5 Department of Ophthalmology, University Medical Center Greifswald, Greifswald, Germany; 6 Eye Center, Medical Center, Faculty of Medicine, University of Freiburg, Freiburg, Germany; 7 Unit of Ophthalmology, Department of Neuroscience, University Hospital, Uppsala, Sweden; 8 Unit of Endocrinology, Department of Physiology, Institute of Neuroscience and Physiology, Sahlgrenska Academy, University of Gothenburg, Gothenburg, Sweden; 9 McKillop Health Institute, Australian Catholic University, Melbourne, Australia; 10 Department of Public Health and Community Medicine, Institute of Medicine, Sahlgrenska Academy, University of Gothenburg, Gothenburg, Sweden; 11 Mathematical Sciences, Chalmers University of Technology, Gothenburg, Sweden; 12 Institute of Biomedicine, Sahlgrenska Academy, University of Gothenburg, Gothenburg, Sweden

## Abstract

**Importance:**

To prevent blindness, repeated infant eye examinations are performed to detect severe retinopathy of prematurity (ROP), yet only a small fraction of those screened need treatment. Early individual risk stratification would improve screening timing and efficiency and potentially reduce the risk of blindness.

**Objectives:**

To create and validate an easy-to-use prediction model using only birth characteristics and to describe a continuous hazard function for ROP treatment.

**Design, Setting, and Participants:**

In this retrospective cohort study, Swedish National Patient Registry data from infants screened for ROP (born between January 1, 2007, and August 7, 2018) were analyzed with Poisson regression for time-varying data (postnatal age, gestational age [GA], sex, birth weight, and important interactions) to develop an individualized predictive model for ROP treatment (called DIGIROP-Birth [Digital ROP]). The model was validated internally and externally (in US and European cohorts) and compared with 4 published prediction models.

**Main Outcomes and Measures:**

The study outcome was ROP treatment. The measures were estimated momentary and cumulative risks, hazard ratios with 95% CIs, area under the receiver operating characteristic curve (hereinafter referred to as AUC), sensitivity, specificity, positive predictive value (PPV), and negative predictive value (NPV).

**Results:**

Among 7609 infants (54.6% boys; mean [SD] GA, 28.1 [2.1] weeks; mean [SD] birth weight, 1119 [353] g), 442 (5.8%) were treated for ROP, including 142 (40.1%) treated of 354 born at less than 24 gestational weeks. Irrespective of GA, the risk for receiving ROP treatment increased during postnatal weeks 8 through 12 and decreased thereafter. Validations of DIGIROP-Birth for 24 to 30 weeks’ GA showed high predictive ability for the model overall (AUC, 0.90 [95% CI, 0.89-0.92] for internal validation, 0.94 [95% CI, 0.90-0.98] for temporal validation, 0.87 [95% CI, 0.84-0.89] for US external validation, and 0.90 [95% CI, 0.85-0.95] for European external validation) by calendar periods and by race/ethnicity. The sensitivity, specificity, PPV, and NPV were numerically at least as high as those obtained from CHOP-ROP (Children’s Hospital of Philadelphia–ROP), OMA-ROP (Omaha-ROP), WINROP (weight, insulinlike growth factor 1, neonatal, ROP), and CO-ROP (Colorado-ROP), models requiring more complex postnatal data.

**Conclusions and Relevance:**

This study validated an individualized prediction model for infants born at 24 to 30 weeks’ GA, enabling early risk prediction of ROP treatment based on birth characteristics data. Postnatal age rather than postmenstrual age was a better predictive variable for the temporal risk of ROP treatment. The model is an accessible online application that appears to be generalizable and to have at least as good test statistics as other models requiring longitudinal neonatal data not always readily available to ophthalmologists.

## Key Points

**Question:**

Can a prediction model be constructed for retinopathy of prematurity needing treatment by using only birth characteristics data and applying advanced statistical methods?

**Findings:**

In this cohort study of 6947 infants born at gestational age 24 to 30 weeks, the prediction model incorporating only postnatal age, gestational age, sex, and birth weight provided a predictive ability for retinopathy of prematurity needing treatment that was comparable to current models requiring postnatal data (not always available). The risk for retinopathy of prematurity needing treatment increased up to 12 weeks’ postnatal age irrespective of the infants’ gestational age.

**Meaning:**

This prediction model identifying infants with a high risk for developing sight-threatening disease at an early time may improve the conditions for optimal screening.

## Teaser

This cohort study creates and validates an easy-to-use prediction model using only birth characteristics and describes a continuous hazard function for retinopathy of prematurity treatment.

## Introduction

Retinopathy of prematurity (ROP) is a potentially blinding disease, and screening programs for detecting sight-threatening ROP needing treatment have been established worldwide.^1,2^ Infants with lower gestational age (GA) have a higher risk of sight-threatening ROP; in Sweden, the recommendation is to screen infants with GA less than 31 weeks and severely ill infants if older. Data are registered in the Swedish National Registry for Retinopathy of Prematurity (SWEDROP). Between 2008 and 2015, only 5.7% of screened infants in Sweden were treated for ROP.^3^ Screening includes retinal examinations by specially trained ophthalmologists and is often stressful for the infant^4^; without risk prediction, some infants may not be screened and treated at the appropriate time. Individualized risk estimates would allow for optimization of timing and frequency of the screening processes from the health care and economics perspectives. Improving the timing of screening visits could avoid unnecessary examinations of low-risk infants and optimize identification of those at high risk.

Risk and severity of ROP vary by prenatal and postnatal factors,^5^ including poor prenatal and postnatal weight gain. For this reason, the prediction algorithm WINROP (weight, insulinlike growth factor 1, neonatal, ROP), which is based on accumulated postnatal weight gain, has been validated and broadly used.^6,7,8,9^ Similar tools based on longitudinal postnatal weight gain also have been developed.^10,11,12,13^ The objectives of this study were to create, then to internally and externally validate, and to describe the clinical implications of a prediction model for individual momentary and cumulative risks of ROP treatment based on birth characteristics alone, including infants born at GA less than 31 weeks.

## Methods

### Study Population

Infants born between January 1, 2007, and August 7, 2018, at GA less than 31 weeks and with completed ROP screening registered in SWEDROP^14^ were included as part of the Swedish Neonatal Quality Register,^15^ started in 2007, which has approximately 97% coverage and contains perinatal data, screening outcomes, and treatment information.^3^ All data are registered through standardized protocols, in most settings by a trained pediatric ophthalmologist who has performed the screening examination. A validation of 85 randomly selected infants screened in 2018 showed 100% correctly reported values for variables used in this study. This retrospective cohort study was approved by the ethics committee at Uppsala University, Uppsala, Sweden, who also waived written informed consent because all the data were deidentified.

#### Model Development Group

**Figure 1.**
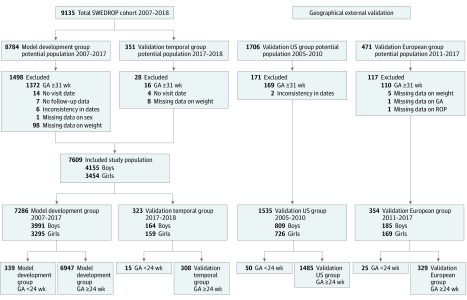
Study Flowchart GA indicates gestational age; ROP, retinopathy of prematurity; and SWEDROP, Swedish National Registry for Retinopathy of Prematurity.

In total, data for 8784 infants born between January 1, 2007, and October 31, 2017, were retrieved from SWEDROP for the prediction model development. Of those, data for 1372 of 8784 infants (15.6%) were excluded for having GA at least 31 weeks at birth, and 126 of 8784 infants (1.4%) were excluded for missing data. This left 7286 of 8784 infants (82.9%) eligible for the model development group. Of those, 6947 of 7286 infants (95.3%) had GA 24 to 30 weeks (Figure 1).

#### Validation Groups

The group used for temporal validation consisted of infants born between November 1, 2017, and August 7, 2018, and registered in SWEDROP. Among infants born at GA 24 to 30 weeks, 308 of 323 (95.4%) were eligible and served as the validation temporal group (Figure 1).

The validation US group included 1485 of 1535 eligible infants (96.7%) born at GA 24 to 30 weeks from 12 US centers between 2005 and 2010 (Figure 1).^16^ The validation European group included 329 of 354 eligible infants (92.9%) born at GA 24 to 30 weeks from Freiburg, Germany, with retrospective screening data collected between 2011 and 2017 (Figure 1).^17^

### Study Procedures

The estimation of GA was based on fetal ultrasonographic results. The chronological (postnatal) age, postmenstrual age, and GA are defined according to the American Academy of Pediatrics’ issued policy.^18^ An SD score (SDS) of expected reference weight (birth weight SDS [BWSDS]) was calculated based on GA, sex, and birth weight for all healthy singletons born at GA at least 24 weeks between 1990 and 1999 in Sweden and registered in the Medical Birth Register (800 000 healthy infants of approximately 1 million born).^19^ Hence, BWSDS was not calculated for infants born at GA less than 24 weeks because of a lack of reference for this extremely preterm population. Infants born at GA less than 24 weeks are at high risk of severe ROP requiring treatment, partly owing to a larger proportion of avascular retinal area at birth,^20^ and prediction models are not as useful in this cohort. Therefore, a simpler prediction model was developed for this group and is presented along with the results in eAppendix 1 (which references eFigures 9-11 and eTables 7 and 8) in the Supplement. Small for GA^19^ was defined as BWSDS less than −2.

### Study Outcome

The prediction model was developed to estimate risk for treatment of sight-threatening ROP. The International Classification of Retinopathy of Prematurity^21^ and Early Treatment for Retinopathy of Prematurity (ETROP)^22^ criteria for treatment were used.

### Statistical Analysis

#### General Methodology

Number and percentage are given for categorical variables; for continuous variables, the mean, SD, median, range, and interquartile range are provided, where applicable. For comparison between 2 groups, we used the Fisher exact test for dichotomous variables, Mantel-Haenszel χ^2^ trend test for ordered categorical variables, and Mann-Whitney test for continuous variables. The Jonckheere-Terpstra test was applied for identifying trends between ordered categorical and continuous variables. The crude week-specific risk of ROP treatment (number of infants with the event divided by number of infants at risk) was analyzed based on postnatal age and postmenstrual age (GA plus postnatal age) by GA at birth. The modeling process consisted of (1) prediction model development, (2) internal and external validation, and (3) clinical implication.^23^ The prediction model for ROP treatment, called DIGIROP-Birth (Digital ROP), was developed using Poisson regression for time-varying data, from which we obtained a continuous hazard function, *h*(*t*), describing momentary risk for ROP treatment.^24,25^ From the hazard function, the survival function 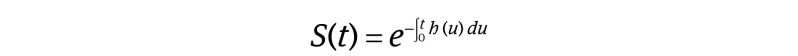
and its complement, the cumulative risk function *F*(*t*) = 1 − *S*(*t*), were estimated. The 95% CI for *F*(*t*) was obtained via repeated sampling (1000 samples) of the model parameters from a multivariate normal distribution using a covariance matrix estimated by the Poisson regression models. Parameter estimates, SEs, and hazard ratios (HRs) with 95% CIs are presented. The predictive ability of the continuous cumulative risks was checked and was found to be similarly high after postnatal age 15 weeks (eFigure 1 in the Supplement). Given this information and the knowledge about the studied hazard function, the cumulative risks of ever needing ROP treatment during 20 postnatal weeks were used for interpretation.

All tests above were 2-tailed and conducted at the .05 significance level, with no adjustments for multiple comparisons. All analyses were performed using SAS statistical software, version 9.4 (SAS Institute Inc).

#### DIGIROP-Birth Prediction Model for GA 24 to 30 Weeks Development and Validation

Based on the crude risks for ROP treatment over time stratified by GA, we found that postnatal age was the most appropriate time axis. The final model for GA 24 to 30 weeks included the following: piecewise linear current postnatal age (break points, 8 and 12 weeks), piecewise linear continuous GA given in weeks and days (break point, 27 weeks), sex, piecewise linear BWSDS (break point, −1 SDS), postnatal age × piecewise linear GA interaction, sex × GA interaction, and postnatal age × piecewise linear BWSDS interaction. The break points for the variables were selected based on graphical review of univariable hazard functions. The final model was built by gradually expanding the models, starting only with postnatal age and further keeping interactions with *P* < .10.

Internal, temporal, and geographical external validations were performed. The model fit and adaptation were described by the area under the receiver operating characteristic curve (hereinafter referred to as AUC) overall, by calendar periods, and by race/ethnicity. We performed cross-validation and evaluated calibration plots; calculated sensitivity, specificity, positive predictive value (PPV), and negative predictive value (NPV); and compared DIGIROP-Birth with 4 other published prediction models (CHOP-ROP [Children’s Hospital of Philadelphia–ROP],^11^ OMA-ROP [Omaha-ROP],^12^ WINROP [weight, insulinlike growth factor 1, neonatal, ROP],^7^ and CO-ROP [Colorado-ROP]^13^) using GA, birth weight, and different weight gain variables in the algorithms, as described in more detail in eAppendix 2 in the Supplement.

## Results

### Study Population

Birth characteristics for the whole SWEDROP cohort, model development group, and validation temporal group, as well as by maximum ROP stage, are listed in eTable 1 in the Supplement. Among 7609 patients, 4155 (54.6%) were boys, the mean (SD) GA was 28.1 (2.1) weeks, and the mean (SD) birth weight was 1119 [353] g. Of those born at GA at least 24 weeks, 1510 of 7255 (20.8%) were small for GA. In total, 354 of 7609 (4.7%) were born at GA less than 24 weeks, and 2806 of 7609 (36.9%) were born at GA 24 to less than 28 weeks. Birth characteristics were numerically balanced between the model development group and the validation temporal group. Birth characteristics for the validation US group and the validation European group are listed in eTable 2 in the Supplement.

### ROP Treatment Incidence in Screened Infants

Altogether, 2427 of 7609 infants (31.9%) developed any ROP, which regressed spontaneously in 1985 of 7609 (26.1%) and was treated in 442 of 7609 (5.8%) (eTable 3 in the Supplement). Among infants with GA less than 24 weeks, 142 of 354 (40.1%) were treated, 287 of 2806 (10.2%) among those with GA 24 to less than 28 weeks and 13 of 4449 (0.3%) among those with GA at least 28 weeks. The incidence of ROP treatment for infants born at GA 24 to 30 weeks was 125 of 1485 (8.4%) in the validation US group and 17 of 329 (5.2%) in the validation European group.

### Momentary Individual Risk of ROP Treatment for GA Less Than 31 Weeks

**Figure 2.**
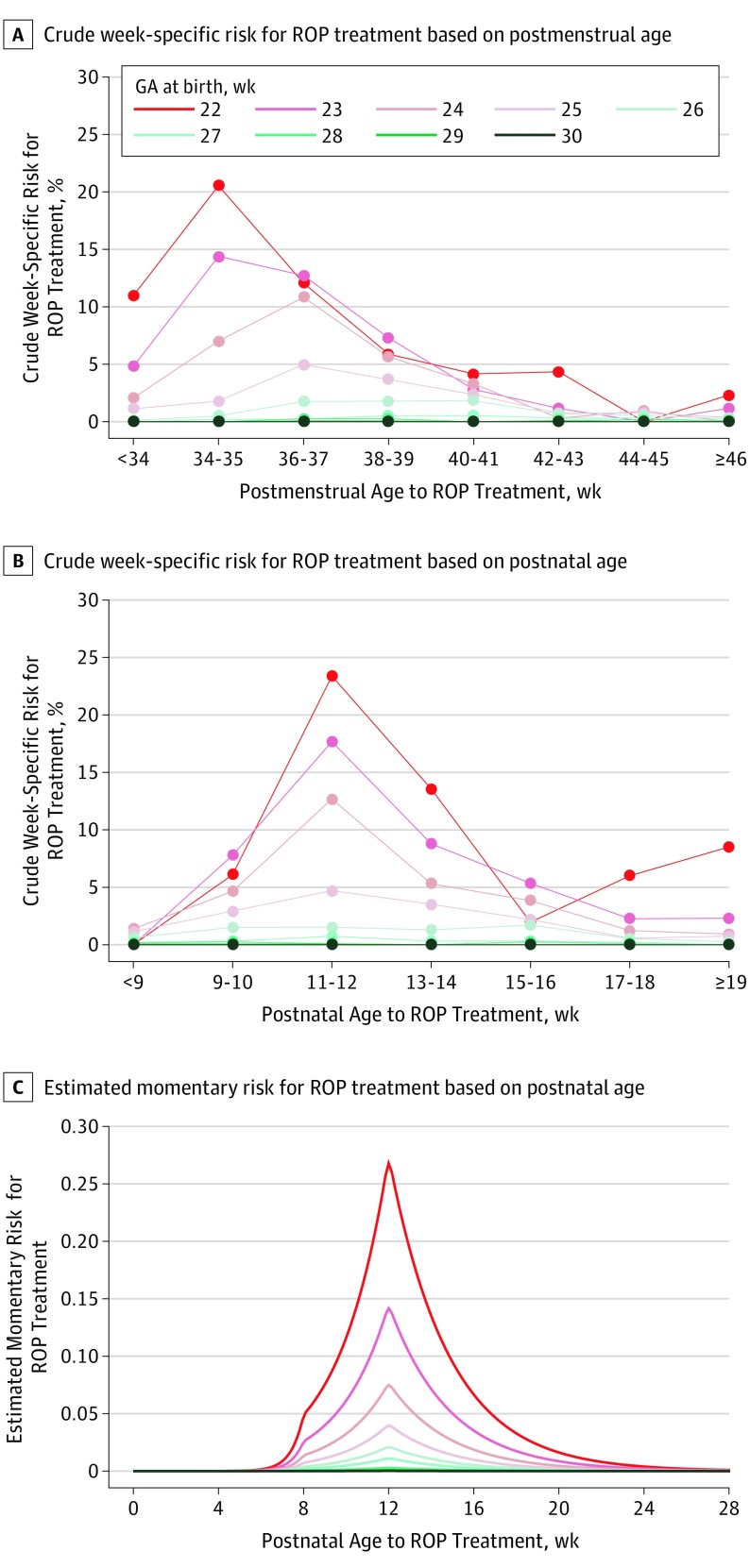
Crude Week-Specific and Momentary Individual Risk of Retinopathy of Prematurity (ROP) Treatment Shown is risk for gestational age (GA) less than 31 weeks.

Figure 2A and B show crude week-specific risk of ROP treatment for the SWEDROP population. Table 1 lists the observed timing for ROP treatment applying postnatal age and postmenstrual age as time axes. The ROP treatment risk peaked at postnatal week 12 regardless of GA at birth, but no specific pattern by GA was seen for postmenstrual age.

**Table 1.** Comparison Between US Guidelines (Fierson et al^2^) Regarding Timing of Initial Examination vs SWEDROP Data and DIGIROP-Birth Model, 2007-2018

GA at Birth, wk	Postmenstrual Age at Initial Examination, wk				Chronological (Postnatal) Age at Initial Examination, wk			
	Fierson et al^2^	SWEDROP 2007-2018 Suggested Age^a^	SWEDROP 2007-2018 Observed Age for ROP Treatment	Maximum Age for Estimated Cumulative Risk <.001^b^	Fierson et al^2^	SWEDROP 2007-2018 Suggested Age^a^	SWEDROP 2007-2018 Observed Age for ROP Treatment	Maximum Age for Estimated Cumulative Risk <.001^b^
21	NR	31	NA	NA	NR	10	NA	NA
Mean (SD)	NA	NA	34.6 (1.7)	NA	NA	NA	12.8 (1.7)	NA
Median (range)	NA	NA	34.6 (33.4-35.9)	NA	NA	NA	12.8 (11.6-14.0)	NA
No./total No.	NA	NA	2/2	NA	NA	NA	2/2	NA
22	31	31	NA	NA	9	9	NA	NA
Mean (SD)	NA	NA	36.3 (3.2)	NA	NA	NA	13.6 (3.2)	NA
Median (range)	NA	NA	35.1 (32.6-47.1)	NA	NA	NA	12.4 (10.0-24.3)	NA
No./total No.	NA	NA	39/82	NA	NA	NA	39/82	NA
23	31	31	NA	NA	8	8	NA	NA
Mean (SD)	NA	NA	36.5 (2.9)	NA	NA	NA	13.1 (2.8)	NA
Median (range)	NA	NA	36.0 (32.9-51.4)	NA	NA	NA	12.6 (9.4-28.3)	NA
No./total No.	NA	NA	101/270	NA	NA	NA	101/270	NA
24	31	31	NA	30.2	7	7	NA	6.2
Mean (SD)	NA	NA	37.1 (2.5)	NA	NA	NA	12.7 (2.5)	NA
Median (range)	NA	NA	36.6 (32.4-45.7)	NA	NA	NA	12.3 (8.3-21.4)	NA
No./total No.	NA	NA	117/436	NA	NA	NA	117/436	NA
25	31	31	NA	31.7	6	6	NA	6.7
Mean (SD)	NA	NA	38.2 (2.9)	NA	NA	NA	12.9 (2.9)	NA
Median (range)	NA	NA	37.7 (33.1-47.0)	NA	NA	NA	12.4 (7.4-21.9)	NA
No./total No.	NA	NA	92/620	NA	NA	NA	92/620	NA
26	31	32	NA	33.2	5	6	NA	7.2
Mean (SD)	NA	NA	39.7 (3.3)	NA	NA	NA	13.3 (3.3)	NA
Median (range)	NA	NA	39.3 (33.1-52.1)	NA	NA	NA	13.0 (7.0-25.6)	NA
No./total No.	NA	NA	58/801	NA	NA	NA	58/801	NA
27	31	33	NA	34.7	4	6	NA	7.7
Mean (SD)	NA	NA	40.3 (2.8)	NA	NA	NA	12.9 (2.9)	NA
Median (range)	NA	NA	40.1 (35.7-45.3)	NA	NA	NA	12.6 (7.9-17.7)	NA
No./total No.	NA	NA	20/949	NA	NA	NA	20/949	NA
28	32	34	NA	35.7	4	6	NA	7.7
Mean (SD)	NA	NA	40.8 (3.8)	NA	NA	NA	12.4 (3.9)	NA
Median (range)	NA	NA	39.4 (36.1-47.7)	NA	NA	NA	11.1 (7.6-18.9)	NA
No./total No.	NA	NA	10/1179	NA	NA	NA	10/1179	NA
29	33	36	NA	37.3	4	7	NA	8.1
Mean (SD)	NA	NA	39.7 (2.2)	NA	NA	NA	10.1 (2.3)	NA
Median (range)	NA	NA	39.4 (37.6-42.0)	NA	NA	NA	9.7 (8.0-12.6)	NA
No./total No.	NA	NA	3/1479	NA	NA	NA	3/1479	NA
30	34	37	NA	38.8	4	7	NA	8.7
No./total No.	NA	NA	0/1791	NA	NA	NA	0/1791	NA

Abbreviations: DIGIROP, Digital ROP; GA, gestational age; NA, not applicable; NR, not reported; ROP, retinopathy of prematurity; SWEDROP, Swedish National Registry for Retinopathy of Prematurity.

^a^ Suggested age defined as integer value of the minimum time to ROP treatment subtracted by 1 week for safety reasons.

^b^ Given the SWEDROP population, DIGIROP-Birth model for GA 24 to 30 weeks, with its sex and birth weight SD score distribution.

From the Poisson regression model based on the total SWEDROP population, including postnatal age and adjusting for GA, the risk for ROP treatment increased by 54% (HR, 1.54; 95% CI, 1.39-1.70) per week from postnatal weeks 8 through 12. Afterward, it decreased by 30% (HR, 0.70; 95% CI, 0.67-0.74) per week (Figure 2C and eTable 4 in the Supplement).

### Cumulative Individual Risk of ROP Treatment for GA 24 to 30 Weeks

**Figure 3.**
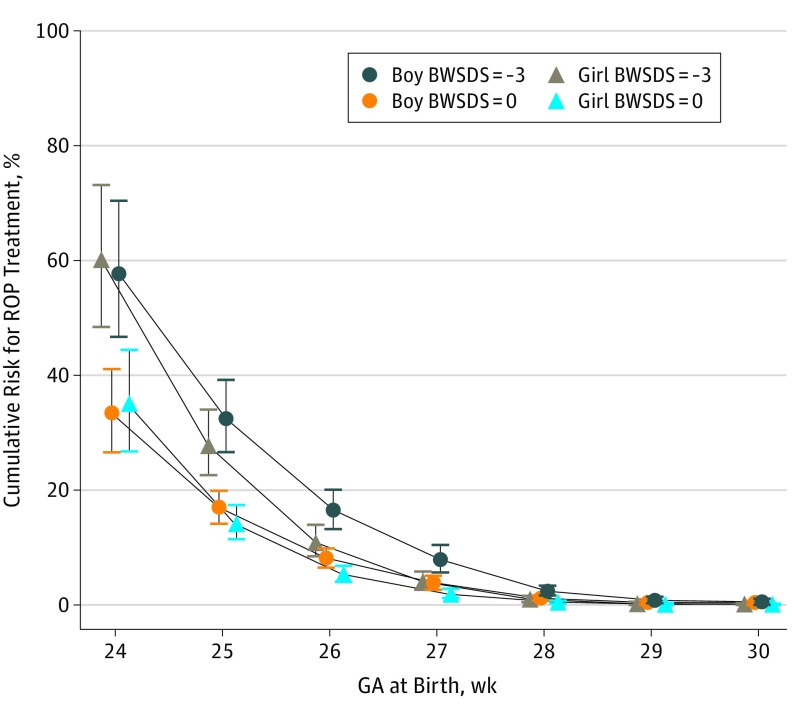
Cumulative Individual Risk for Retinopathy of Prematurity (ROP) Treatment Shown is cumulative risk (95% CI) by gestational age (GA) 24 to 30 weeks for boys and girls born with birth weight SD score (BWSDS) −3 and 0.

Table 2 summarizes the final DIGIROP-Birth model for ROP treatment in infants born at GA 24 to 30 weeks. The estimated cumulative risks were 60.0% and 35.1%, respectively, for a girl with BWSDS −3 and 0 born at GA 24 weeks and were 27.8% and 14.2%, respectively, if she was born at GA 25 weeks (Figure 3 and eFigure 2 and eTable 5 in the Supplement). Corresponding figures for a boy with the same background data were 57.7% and 33.4%, respectively, and 32.5% and 16.9%, respectively. Greater decreasing risk was observed for girls than for boys with increasing GA (*P* for interaction = .02), with HRs of 0.83 (95% CI, 0.64-1.07) at 25 weeks and 0.50 (95% CI, 0.33-0.76) at 27 weeks (crude incidences are shown in eFigure 3 in the Supplement, and predicted cumulative risks are shown in eFigure 4 in the Supplement). The cumulative risk estimates with 95% CIs are available online for public use,^26^ requiring input of GA in weeks and days, sex, and birth weight for the infant.

**Table 2.** Final Prediction Analysis Model for Retinopathy of Prematurity Treatment for Infants Born at GA of 24 to 30 Weeks Using Poisson Regression for Time-Varying Data

Predictor	Estimate (SE)	*P* Value
Intercept	−20.1666 (4.9219)	<.001
Postnatal age 0 to 8 wk, per 1-wk increase	1.7331 (0.6129)	.005
Postnatal age >8 to 12 wk, per 1-wk increase	0.3618 (0.0992)	<.001
Postnatal age >12 wk, per 1-wk increase	−0.3788 (0.0857)	<.001
GA 24-27 wk, per 1-wk increase	−0.8210 (0.3353)	.01
GA >27 wk, per 1-wk increase	0.7266 (0.7302)	.32
Sex, 1 = boys, 2 = girls	−0.9385 (0.3054)	.002
BWSDS −1 SDS or less, per 1-SDS increase	0.1521 (0.2656)	.57
BWSDS exceeding −1 SDS, per 1-SDS increase	−1.0401 (0.4710)	.03
INT: postnatal age in weeks by GA 24-27 wk, per 1-wk increase	0.0227 (0.0230)	.32
INT: postnatal age in weeks by GA >27 wk, per 1-wk increase	−0.1360 (0.0627)	.03
INT: sex by GA, per 1-wk increase	−0.2505 (0.1066)	.02
INT: postnatal age in weeks by BWSDS −1 SDS or less	−0.0371 (0.0199)	.06
INT: postnatal age in weeks by BWSDS exceeding −1 SDS	0.0728 (0.0349)	.04

Abbreviations: BWSDS, birth weight standard deviation score; GA, gestational age; INT, interaction term; SDS, SD score.

### Internal and External Validation of DIGIROP-Birth for GA 24 to 30 Weeks

eFigure 5 in the Supplement shows AUCs from the internal and external validations, indicating whether the model discriminates well between receiving or not receiving treatment. The AUC for the model development group was 0.90 (95% CI, 0.89-0.92), and the AUC for the cross-validation model was 0.90 (95% CI, 0.89-0.91). The AUCs for different calendar periods ranged from 0.87 to 0.92. The calibration plots, examining overestimation or underestimation of risks in different regions, showed the model as being overall well adapted (eFigure 6 in the Supplement). Temporal validation of DIGIROP-Birth showed an AUC of 0.94 (95% CI, 0.90-0.98). Geographical external validation resulted in an AUC of 0.87 (95% CI, 0.84-0.89) for the validation US group and an AUC of 0.90 (95% CI, 0.85-0.95) for the validation European group. The AUCs for stratified analysis on race/ethnicity categories in the validation US group were 0.79 for Hispanic infants, 0.85 for Asian infants, 0.86 for non-Hispanic infants, 0.88 for white infants, and 0.90 for black infants.

### DIGIROP-Birth for GA 24 to 30 Weeks vs Existing ROP Models (Requiring Postnatal Longitudinal Data)

The comparisons of DIGIROP-Birth vs CHOP-ROP, OMA-ROP, WINROP, and CO-ROP were performed on the validation US group, enabling the use of longitudinal weight data. These results are summarized in eFigures 7 and 8 and eTable 6 in the Supplement.

Applying the CHOP-ROP algorithm (AUC, 0.89; 95% CI, 0.87-0.92) and categorizing the probabilities based on the recommended cutoff of 0.0140, similar prediction ability was observed compared with DIGIROP-Birth (AUC, 0.88; 95% CI, 0.86-0.91), and a cutoff of 0.0083 obtained the same sensitivity (95 of 96 [99.0%] for both CHOP-ROP and DIGIROP-Birth. Specificity was 598 of 1346 (44.4%) vs 658 of 1346 (48.9%), respectively. Applying the same cutoff on the complete SWEDROP database, the model showed 97.7% (95% CI, 95.3%-99.1%) sensitivity and 59.5% (95% CI, 58.4%-60.7%) specificity. Applying a cutoff of 0.00083 for 100% (95% CI, 98.8%-100%) sensitivity in the cohort, a specificity of 19.0% (95% CI, 18.1%-20.0%) was obtained.

Compared with OMA-ROP (AUC, 0.77; 95% CI, 0.72-0.82), a cutoff of 23 g per day in weight gain, with a corresponding cutoff of 0.0200 for DIGIROP-Birth (AUC, 0.90; 95% CI, 0.87-0.92), a sensitivity of 90 of 92 (97.8%) was obtained. Specificity was 173 of 771 (22.4%) for OMA-ROP vs 448 of 771 (58.1%) for DIGIROP-Birth.

Compared with WINROP (AUC, 0.81; 95% CI, 0.78-0.84), the alarm category of WINROP score 2 or 3 provided a sensitivity of 121 of 125 (96.8%), with a corresponding cutoff of 0.0089 for DIGIROP-Birth (AUC, 0.87; 95% CI, 0.84-0.89). Specificity was 487 of 1360 (35.8%) for WINROP vs 671 of 1360 (49.3%) for DIGIROP-Birth.

The specificity was 141 of 1341 (10.5%) for the CO-ROP algorithm and 642 of 1341 (47.9%) for DIGIROP-Birth. Both had a sensitivity of 122 of 124 (98.4%).

### Clinical Practice Implications of DIGIROP-Birth for GA 24 to 30 Weeks

Based on ROP treatment timing in the SWEDROP cohort (2007-2018) and the DIGIROP-Birth model, we compared the results with the current US recommendations, based on studies that are more than 20 years old,^2^ for postnatal age and postmenstrual age at initial examination (Table 1). The maximum age for estimated risk less than 0.001 corresponds well to the observed minimum age for ROP treatment, except for GA 24 weeks, for which a somewhat higher risk at a younger age was estimated. Recommending that the initial examination should start 1 week before the earliest observed ROP treatment per GA week in our cohort would potentially have avoided 14 867 of 135 061 visits (11.0%), assuming 1 visit per week. For GA of at least 27 weeks, with a ROP treatment incidence of 33 of 5398 (0.6%), the difference between the US recommendations and this study resulted in 14 066 of 93 052 examinations (15.1%) potentially being avoided.

## Discussion

We have created and validated the DIGIROP-Birth prediction model, available free of charge online^26^ based on 6947 infants born at GA 24 to 30 weeks, estimating the individual momentary and cumulative risks for ROP treatment. The model using only available data at birth but more advanced statistical methods was at least as accurate as 4 of the ROP prediction models now in use based on longitudinal weight measurements, which are not always readily available to ophthalmologists.

Surprisingly, the momentary risk of ROP treatment peaked at 12 weeks’ postnatal age regardless of GA at birth, while no specific pattern was observed for postmenstrual age. This observation is particularly interesting because the ETROP study^27^ found that the progression of prethreshold ROP was highly associated with postmenstrual age, similar to the finding in the CRYO-ROP (Cryotherapy for ROP) study^28^ 15 years earlier. However, it should be emphasized that infants included in the CRYO-ROP study were born at higher GA, and no GA-specific hazard functions were studied for ROP outcome. Other Swedish studies^29,30^ have reported that lower GA at birth is associated with lower GA at treatment, but the momentary risk in relation to postnatal age and postmenstrual age was not analyzed. Recently, in a large North American cohort, the timing of ROP treatment was presented only in relation to postmenstrual age and not postnatal age.^31^

The identification of a peak risk at 12 postnatal weeks in infants with GA less than 31 weeks might be clinically useful because it was recently shown that inadequate screening or treatment was identified in 11 of 17 cases with blindness from ROP (64.7%).^32^ Hence, clinicians and parents could be alerted during this period to ensure that timely screening occurs to reduce the risk of blindness.

National patient registries are valuable sources for estimation of treatment risks. Herein, the DIGIROP-Birth model was compared with a validation US group and a validation European group and showed high predictive ability and generalizability both for individuals with the same and with different reported race/ethnicity.

The ROP prediction models may also be used to reduce screening frequency in infants at low risk. The latest US policy statement for ROP screening^2^ was issued in 2018. The recommendations for the timing of the first examination were based on the CRYO-ROP study^28^ published in 1991 and the LIGHT-ROP (Light Reduction in ROP) study^33^ published in 1998. In those periods, fewer extremely preterm infants survived, more mature infants were treated, and treatment criteria were different from those used today. Based on the results of our study, if the initial examination was performed 1 week before the earliest observed postnatal age at ROP treatment, 14 867 of 135 061 stressful early examinations (11.0%) could be avoided (assuming 1 examination per week) compared with US recommendations.^2^ For GA of at least 27 weeks, with a ROP treatment incidence herein less than 1%, 14 066 of 93 052 examinations (15.1%) could have been avoided while capturing all cases of ROP treatment (100% sensitivity). Notably, reaching 100% sensitivity in such models of real-life, large data sets is accompanied by low specificity. Based on approximately as large a cohort as in our study, the updated CHOP-ROP^11^ model, which uses longitudinal weight data and birth data, achieved 11.2% specificity for 100% sensitivity and 36.4% specificity for 98.5% sensitivity; DIGIROP-Birth (using only readily obtained birth data) showed 19.0% specificity for 100% sensitivity and 53.8% specificity for 99.0% sensitivity.

### Strengths and Limitations

The strengths of our study include the unique and complete cohort of preterm infants born in Sweden between January 2007 and August 2018. Also, our statistical model includes 3 basic measurements (GA, sex, and birth weight). The postnatal age for ROP treatment or censoring (discontinued follow-up) is included in the hazard function estimation but is not required as an input variable. Hence, the input data are simple, facilitating their general use, even though the method is more advanced, taking into account the underlying hazard function and the important interactions that contribute to adjustment of heterogeneity, which is novel in ROP research. The DIGIROP-Birth has shown strong predictive ability in internal, temporal, and geographical external validations. If found not acceptable in future validations among a population, a subgroup-specific model designed for optimal predictions in that population might be developed using our methods. Finally, DIGIROP-Birth has been shown to be equal to or better than 4 other ROP prediction models and is accessible online.^26^

Our study has some limitations. One limitation is the use of registry retrospective data. However, the registry showed high coverage and successful validation of data for 85 randomly selected infants screened in 2018. In addition, infants born at GA less than 24 weeks could not be included in the prediction model because of the lack of a reference algorithm for birth weight, preventing BWSDS calculations. Given the small sample size, only a simple model could be developed for these infants, resulting in low predictive ability. Close monitoring of such infants is mandatory irrespective of calculated risk, making prediction models less important for this group.

## Conclusions

We created and validated the DIGIROP-Birth model, an individualized early prediction model for infants with GA 24 to 30 weeks, which estimates momentary and cumulative risks for receiving ROP treatment based on simple birth characteristics. A surprising finding was that postnatal age was the best predictive variable for the temporal risk of ROP treatment. The DIGIROP-Birth model is an accessible online application that appears to be generalizable and to have at least as good test statistics as other models that require longitudinal neonatal data, which are not always readily available to ophthalmologists.

## Notes

Supplement. **eAppendix 1.** Prediction Model for Gestational Age <24 Weeks **eAppendix 2.** Internal and External Validation **eFigure 1.** AUC for DIGIROP-Birth Model Performed on Model Group for Cumulative Probabilities Estimated Over the Postnatal Age **eFigure 2.** Momentary and Cumulative Individual Risk With 95% CI Over Time for ROP Treatment for Gestational Age 24 and 25 Weeks, by Sex for Different Birth WeightSDS **eFigure 3.** Incidence of ROP Treatment by Gestational Age, Sex, and Birth WeightSDS (BWSDS) **eFigure 4.** Estimated Risk (%) for ROP Treatment by Gestational Age and Sex **eFigure 5.** ROC Curves Obtained Based on Cumulative Individual Risk for ROP Treatment for Gestational Age at Birth ≥24 Weeks From Model Group, Validation Temporal Group, Validation US Group and Validation European Group (A), by Calendar Periods in the Model Group (B), and by Race/Ethnicity in the Validation US Group (C) **eFigure 6.** Calibration Plot for Observed Proportion of ROP Treatment vs Estimated Probability Obtained From the Final Prediction Model for Gestational Age at Birth ≥24 Weeks (A), and From Cross-Validation Model for Gestational Age ≥24 Weeks (B) **eFigure 7.** ROC Curves Obtained Based on Cumulative Individual Risk for ROP Treatment for Gestational Age at Birth ≥24 Weeks From Validation US Group. DIGIROP-Birth vs CHOP-ROP (A), DIGIROP-Birth vs OMA-ROP (B), DIGIROP-Birth vs WINROP (C), and DIGIROP-Birth vs CO-ROP (D) **eFigure 8.** Scatterplots for Individual Risk Predictions for ROP Treatment Performed on Validation US Group, Obtained From DIGIROP-Birth and CHOP-ROP Models (A), DIGIROP-Birth and CHOP-ROP Models Zoom-in Figure for Probabilities 0.0-0.1 (B), DIGIROP-Birth and OMA-ROP Models (C), and DIGIROP-Birth and WINROP Models (D). **eFigure 9.** Cumulative Individual Risk With 95% CI for ROP Treatment for Gestational Age at Birth <24 Weeks **eFigure 10.** ROC Curves Obtained Based on Cumulative Individual Risk for ROP Treatment for Gestational Age at Birth <24 Weeks From the Main Study, Cross-Validation, and External Population Database (A) and by Calendar Periods for the Main Study Database (B) **eFigure 11.** Calibration Plot for Observed Proportion of ROP Treatment vs Estimated Probability Obtained From the Final Prediction Model for Gestational Age at Birth <24 Weeks (A), and From the Cross-Validation Model for Gestational Age <24 Weeks (B) **eTable 1.** Birth Characteristics for Total SWEDROP Cohort by Study Population and by Maximum ROP Stage **eTable 2.** Birth Characteristics for Validation US Group and Validation European Group **eTable 3.** Number, Percentage, and Follow-up Weeks for ROP Treatment, by Sex, Gestational Age at Birth, and Birth WeightSDS **eTable 4.** Prediction Models for ROP Treatment for Total SWEDROP Cohort Using Only Postnatal Age and Gestational Age – Poisson Regression for Time-Varying Data **eTable 5.** Estimated Probability for ROP Treatment With 95% CI for Selected Values of Birth WeightSDS, Gestational Age at Birth, Sex, and Postnatal Age – Final Poisson Regression Model for Gestational Age at Birth ≥24 Weeks **eTable 6.** DIGIROP-Birth vs CHOP-ROP, OMA-ROP, WINROP and CO-ROP. AUC, Sensitivity, Specificity, PPV and NPV **eTable 7.** Final Prediction Model for ROP Treatment for Infants With Gestational Age at Birth <24 Weeks – Poisson Regression for Time-Varying Data **eTable 8.** Estimated Probability for ROP Treatment With 95% CI for Selected Values of Birth Weight, Gestational Age at Birth, Sex, and Postnatal Age – Final Poisson Regression Model for Gestational Age at Birth <24 Weeks **eReferences**
