# Supplementary material for: Individual Risk Prediction for Sight-Threatening Retinopathy of Prematurity Using Birth Characteristics
Source: JAMA Ophthalmol. 2019 Nov 7;138(1):21–9. doi: 10.1001/jamaophthalmol.2019.4502 (PMC6865304; doi:10.1001/jamaophthalmol.2019.4502)
Supplement: Supplement. — eAppendix 1. Prediction Model for Gestational Age <24 Weeks eAppendix 2. Internal and External Validation eFigure 1. AUC for DIGIROP-Birth Model Performed on Model Group for Cumulative Probabilities Estimated Over the Postnatal Age eFigure 2. Momentary and Cumulative Individual Risk With 95% CI Over Time for ROP Treatment for Gestational Age 24 and 25 Weeks, by Sex for Different Birth WeightSDS eFigure 3. Incidence of ROP Treatment by Gestational Age, Sex, and Birth WeightSDS (BWSDS) eFigure 4. Estimated Risk (%) for ROP Treatment by Gestational Age and Sex eFigure 5. ROC Curves Obtained Based on Cumulative Individual Risk for ROP Treatment for Gestational Age at Birth ≥24 Weeks From Model Group, Validation Temporal Group, Validation US Group and Validation European Group (A), by Calendar Periods in the Model Group (B), and by Race/Ethnicity in the Validation US Group (C) eFigure 6. Calibration Plot for Observed Proportion of ROP Treatment vs Estimated Probability Obtained From the Final Prediction Model for Gestational Age at Birth ≥24 Weeks (A), and From Cross-Validation Model for Gestational Age ≥24 Weeks (B) eFigure 7. ROC Curves Obtained Based on Cumulative Individual Risk for ROP Treatment for Gestational Age at Birth ≥24 Weeks From Validation US Group. DIGIROP-Birth vs CHOP-ROP (A), DIGIROP-Birth vs OMA-ROP (B), DIGIROP-Birth vs WINROP (C), and DIGIROP-Birth vs CO-ROP (D) eFigure 8. Scatterplots for Individual Risk Predictions for ROP Treatment Performed on Validation US Group, Obtained From DIGIROP-Birth and CHOP-ROP Models (A), DIGIROP-Birth and CHOP-ROP Models Zoom-in Figure for Probabilities 0.0-0.1 (B), DIGIROP-Birth and OMA-ROP Models (C), and DIGIROP-Birth and WINROP Models (D). eFigure 9. Cumulative Individual Risk With 95% CI for ROP Treatment for Gestational Age at Birth <24 Weeks eFigure 10. ROC Curves Obtained Based on Cumulative Individual Risk for ROP Treatment for Gestational Age at Birth <24 Weeks From the Main Study, Cross-Validation, and Ext [file jamaophthalmol-138-21-s001.pdf]

Supplementary Online Content

Pivodic A, Hård A-L, Löfqvist C, et al. Individual risk prediction for sight-threatening retinopathy of prematurity using birth characteristics. *JAMA Ophthalmol*. Published online November 7, 2019. doi:10.1001/jamaophthalmol.2019.4502

eAppendix 1. Prediction Model for Gestational Age <24 Weeks

eAppendix 2. Internal and External Validation

eFigure 1. AUC for DIGIROP-Birth Model Performed on Model Group for Cumulative Probabilities Estimated Over the Postnatal Age

eFigure 2. Momentary and Cumulative Individual Risk With 95% CI Over Time for ROP Treatment for Gestational Age 24 and 25 Weeks, by Sex for Different Birth Weight$_{SDS}$

eFigure 3. Incidence of ROP Treatment by Gestational Age, Sex, and Birth Weight$_{SDS}$ (BWSDS)

eFigure 4. Estimated Risk (%) for ROP Treatment by Gestational Age and Sex

eFigure 5. ROC Curves Obtained Based on Cumulative Individual Risk for ROP Treatment for Gestational Age at Birth ≥24 Weeks From Model Group, Validation Temporal Group, Validation US Group and Validation European Group (A), by Calendar Periods in the Model Group (B), and by Race/Ethnicity in the Validation US Group (C)

eFigure 6. Calibration Plot for Observed Proportion of ROP Treatment vs Estimated Probability Obtained From the Final Prediction Model for Gestational Age at Birth ≥24 Weeks (A), and From Cross-Validation Model for Gestational Age ≥24 Weeks (B)

eFigure 7. ROC Curves Obtained Based on Cumulative Individual Risk for ROP Treatment for Gestational Age at Birth ≥24 Weeks From Validation US Group. DIGIROP-Birth vs CHOP-ROP (A), DIGIROP-Birth vs OMA-ROP (B), DIGIROP-Birth vs WINROP (C), and DIGIROP-Birth vs CO-ROP (D)

eFigure 8. Scatterplots for Individual Risk Predictions for ROP Treatment Performed on Validation US Group, Obtained From DIGIROP-Birth and CHOP-ROP Models (A), DIGIROP-Birth and CHOP-ROP Models Zoom-In Figure for Probabilities 0.0-0.1 (B), DIGIROP-Birth and OMA-ROP Models (C), and DIGIROP-Birth and WINROP Models (D).

eFigure 9. Cumulative Individual Risk With 95% CI for ROP Treatment for Gestational Age at Birth <24 Weeks

eFigure 10. ROC Curves Obtained Based on Cumulative Individual Risk for ROP Treatment for Gestational Age at Birth <24 Weeks From the Main Study, Cross-Validation, and External Population Database (A) and by Calendar Periods for the Main Study Database (B)

eFigure 11. Calibration Plot for Observed Proportion of ROP Treatment vs Estimated Probability Obtained From the Final Prediction Model for Gestational Age at Birth <24 Weeks (A), and From the Cross-Validation Model for Gestational Age <24 Weeks (B)

eTable 1. Birth Characteristics for Total SWEDROP Cohort by Study Population and by Maximum ROP Stage

eTable 2. Birth Characteristics for Validation US Group and Validation European Group

eTable 3. Number, Percentage, and Follow-up Weeks for ROP Treatment, by Sex, Gestational Age at Birth, and Birth Weight$_{SDS}$

eTable 4. Prediction Models for ROP Treatment for Total SWEDROP Cohort Using Only Postnatal Age and Gestational Age—Poisson Regression for Time-Varying Data

eTable 5. Estimated Probability for ROP Treatment With 95% CI for Selected Values of Birth Weight$_{SDS}$, Gestational Age at Birth, Sex, and Postnatal Age—Final Poisson Regression Model for Gestational Age at Birth ≥24 Weeks

eTable 6. DIGIROP-Birth vs CHOP-ROP, OMA-ROP, WINROP and CO-ROP. AUC, Sensitivity, Specificity, PPV and NPV

eTable 7. Final Prediction Model for ROP Treatment for Infants With Gestational Age at Birth<24 Weeks—Poisson Regression for Time-Varying Data

eTable 8. Estimated Probability for ROP Treatment With 95% CI for Selected Values of Birth Weight, Gestational Age at Birth, Sex, and Postnatal Age—Final Poisson Regression Model for Gestational Age at Birth <24 Weeks

eReferences

This supplementary material has been provided by the authors to give readers additional information about their work.

eAppendix 1. Prediction Model for Gestational Age <24 Weeks

Methods

Statistical Analysis

Prediction Model Development and Validation

Based on the obtained crude risks for ROP treatment over time by different GAs at birth, we concluded that the most appropriate time axis was postnatal age. The final model for GA<24 weeks included piecewise-linear postnatal age with a break point at 12 weeks, continuous GA, sex, and piecewise-linear birth weight (breakpoint at 550 g).

Hazard functions for one variable at a time were graphically reviewed when selecting the break points for the continuous variables. The final models were built starting with a simple model including only postnatal age and gradually expanding with available variables and interactions between them.

Internal and external validations of the prediction model were performed. The model fit and adaption were described by area under the receiver operating characteristic (ROC) curve (AUC) and by evaluating calibration plots, described in Supplementary Appendix 2.

Results

Cumulative Individual Risk for ROP treatment

eTable 7, eTable 8, and eFigure 9 in the Supplement show the results from the final model obtained for infants born at GA<24 weeks. The effects of GA, sex, and birth weight ≤550 g were not significant. However, there was a decrease in risk for ROP treatment by 20% (HR, 0.80 [95% CI, 0.66–0.97], P=0.03) for each 50-g increase in birth weight >550 g.

Internal and External Validation

The AUCs for infants born at GA<24 weeks were not acceptable at 0.59 (95% CI, 0.53–0.65) using the main model probabilities and 0.55 (95% CI, 0.49–0.61) using the ones from the cross-validation (eFigure 10 in the Supplement). eFigure 11 in the Supplement presents calibration plots obtained for the model.

eAppendix 2. Internal and External Validation

Internal Validation

The two models were validated using a 10-fold cross-validation method by randomly subdividing the dataset into 10 equally large subsets. Each $1/10^{th}$ subset is used as *validation* data based on the coefficients obtained from the *training* $9/10^{th}$ subset. In this way, each observation is used only once for *validation* and nine times for *training*. Calibration plots were produced showing observed proportions with exact 95% CIs for ROP treatment versus mean estimated probability from the cross-validation models in categories (<0.30, 0.30–<0.35, 0.35–<0.40, 0.40–<0.45, 0.45–<0.50, and ≥0.50 for the GA<24 weeks model; <0.05, 0.05–<0.10, 0.10–<0.15, 0.15–<0.20, 0.20–<0.25, 0.25–<0.30, and ≥0.30 for GA ≥24 weeks model) of the individual estimated probability, and the regression spline was fitted to identify underestimation or overestimation of the calibration in specific regions. For comparison, similar calibration plots were made using the estimated probabilities from the main Poisson model overall and by calendar periods (2007–2008, 2009-2010, 2011–2012, 2013–2014, 2015–2017) to validate predictive ability over time. In addition, ROC curves were constructed with ROP treatment as outcome and each individual estimated probability from the cross-validation and the main mode on *Model Group* as the explanatory variable, and the AUCs were numerically compared. An AUC of 0.5 means that the model is not predicting better than any other randomly selected model, while a value of 1 means that the model is discriminating the infants with and without events correctly at every unique value of the predictor(s). An AUC between 0.7–<0.8 is considered as acceptable, 0.8–<0.9 as excellent, and ≥0.9 outstanding.[1]

External Validation

The parameter estimates obtained from the main model for GA 24–30 weeks were applied onto the *Validation Temporal Group*, *Validation US Group* and *Validation European Group*, and risks for ROP treatment were estimated. The ROC curves were produced and numerically compared to the ones obtained from the internal validation.

External validation was also performed comparing the prediction ability of the DIGIROP-Birth model directly to three of the broadly used ROP prediction models, CHOP-ROP[2], OMA-ROP[3], and WINROP[4]. All of the three models are using longitudinal weight data implying that this validation was possible to be performed on the *Validation US Group* where weekly weights are available for infants. The AUC, sensitivity, specificity, positive predicted value (PPV) and negative predicted value (NPV) were calculated based on calculations and cut-offs specified below.

Comparisons versus CHOP-ROP

The probability for ROP treatment = 1/[1+exp(-risk score)] where

Risk score = (-1.50) + (4.24 if GA=23) + (3.49 if GA=24) + (3.60 if GA=25) + (2.33 if GA=26) + (2.48 if GA=27) + [(-0.0037) × (BW)] + [(-0.0186) × (weight gain rate)], where weight gain rate was calculated as [(mean of daily weights of the preceding week – mean of daily weights of the penultimate week)/7].

Following assumptions and restrictions were made regarding the data:

1. Include only patients with GA 24-<31 weeks
2. For all GA > 28 weeks assume the GA parameter is 0, otherwise 3.49 used for GA=24, 3.60 for GA=25 etc according to the algorithm above
3. For patients receiving ROP treatment:
   a. Require the latest weekly weight to be reported at most 8 days before the ROP treatment date
   b. Require the penultimate weekly weight to be reported at latest 8 days before the latest weekly weight
   c. If any of the weights above are not available then the score is not calculated and patient not included in either of the algorithm calculations
   d. If both weights as per above are available then calculate the weight gain rate as ($weight_{PRECEDING}$ – $weight_{PENULTIMATE}$)/7.
4. For patients not receiving ROP treatment:
   a. Calculate the weight gain rate for the LAST and PENULTIMATE weight available in the database and require not more than 8 days in-between the two lastly collected weekly weights.
5. The cut-off of 0.0034 and 0.0140 were used for calculation of sensitivity, specificity, PPV and NPV. The cut-off corresponding to the same sensitivity for DIGIROP-Birth model was 0.0076 and 0.0083 and sensitivity was 100% and 99.0% respectively.

Comparisons versus OMA-ROP

Following assumptions and restrictions were made regarding the data:

1. Include only patients with GA 24-<31 weeks
2. For patients receiving ROP treatment before postmenstrual age of 36 weeks select the latest weight available before 36.5 postmenstrual weeks and before ROP treatment
3. For patients receiving ROP treatment after postmenstrual age of 36 weeks or not receiving ROP treatment select the latest weight available for the period 35.5-36.5 weeks
4. Calculate the OMA-ROP score according to following algorithm: (selected latest weight as per above - BW)/(date for latest weight – date of birth+1)
5. The cut-off of 23 grams/day was used for calculation of sensitivity, specificity, PPV and NPV. The cut-off corresponding to the same sensitivity for DIGIROP-Birth model was 0.0200 and sensitivity was 97.8%.

Comparisons versus WINROP

The database and already calculated WINROP risk score of [0,1,2,3] used in the Wu et al[5] publication was used in the comparison versus DIGIROP-Birth. The cut-off [2,3] vs [0,1] was applied for calculation of sensitivity, specificity, PPV and NPV. The cut-off corresponding to the same sensitivity for DIGIROP-Birth model was 0.0089 and sensitivity was 96.8%.

Comparisons versus CO-ROP

Following assumptions and restrictions were made regarding the data:

1. Include only patients with GA 24-<31 weeks
2. Calculate the CO-ROP alarm to equal 1 for infants with GA<31 weeks and birth weight ≤1500 gram and weight gain from birth to postnatal week 4 ≤650 gram, otherwise if all three parameters are non-missing than CO-ROP alarm should be equal to 0.
3. The cut-off of 1 vs 0 for the alarm variable was used for calculation of sensitivity, specificity, PPV and NPV. The cut-off corresponding to the same sensitivity for DIGIROP-Birth model was 0.0076 and sensitivity was 98.4%.

eFigure 1. AUC for DIGIROP-Birth Model Performed on Model Group for Cumulative Probabilities Estimated Over the Postnatal Age

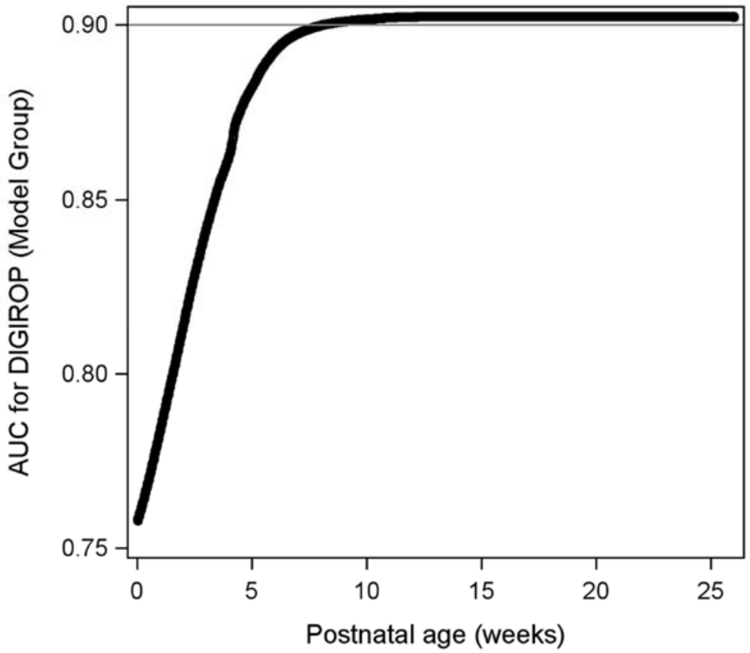

eFigure 2. Momentary and Cumulative Individual Risk with 95% CI over Time for ROP Treatment for Gestational Age 24 and 25 Weeks, by Sex for Different Birth Weight$_{SDS}$[3]

A) Girls – GA 24 weeks – Momentary

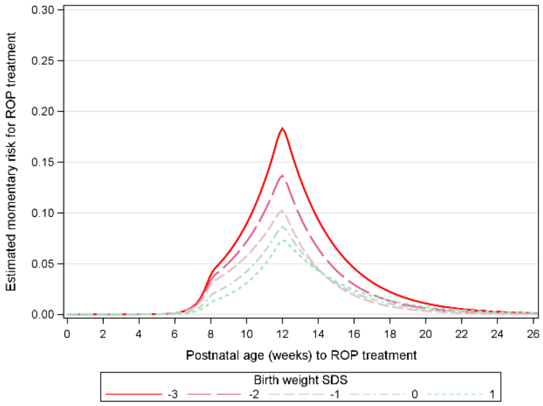

B) Girls – GA 24 weeks – Cumulative

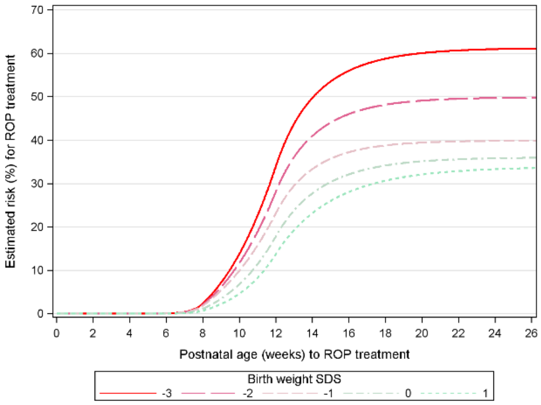

C) Boys – GA 24 weeks – Momentary

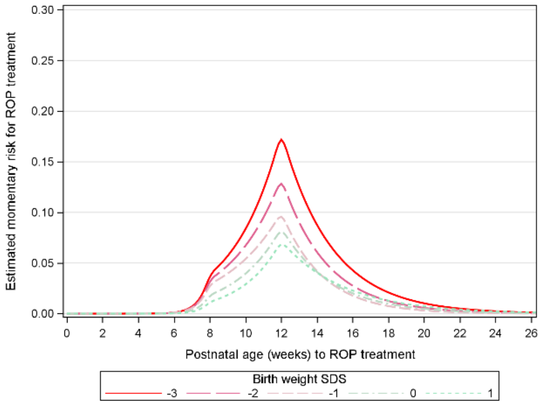

D) Boys – GA 24 weeks – Cumulative

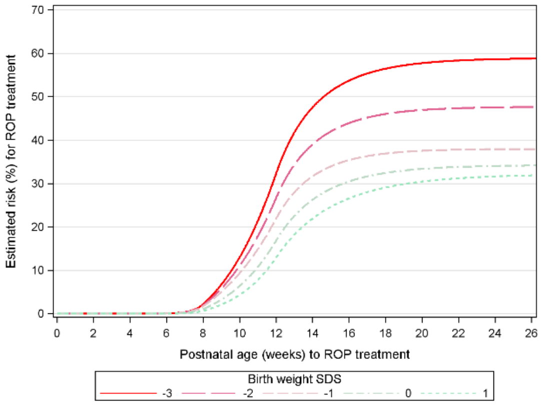

E) Girls – GA 25 weeks – Momentary

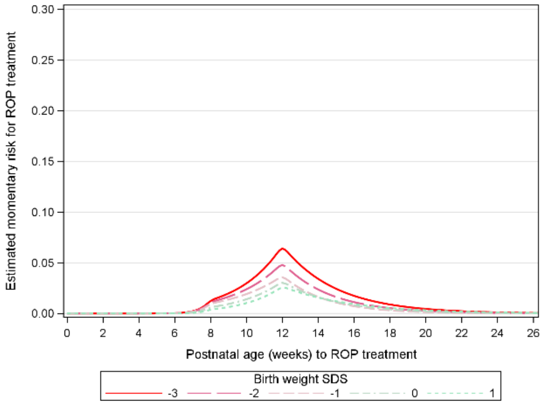

F) Girls – GA 25 weeks – Cumulative

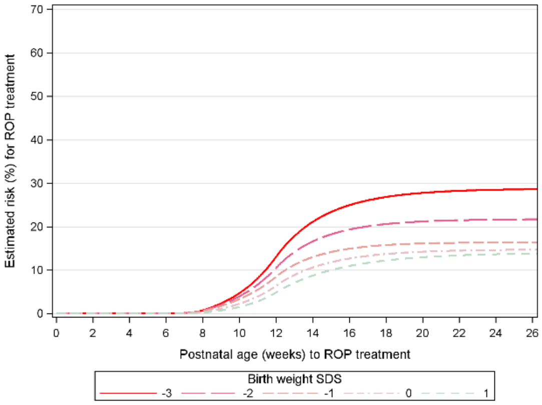

G) Boys – GA 25 weeks – Momentary

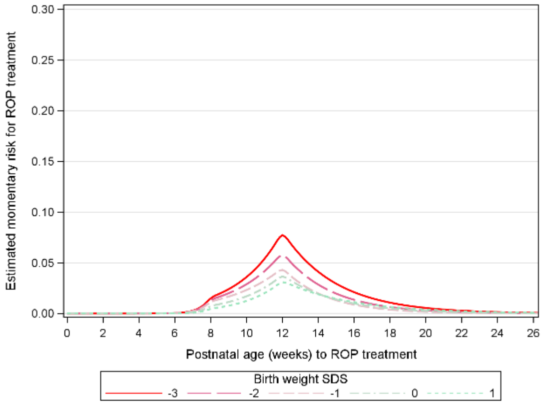

H) Boys – GA 25 weeks – Cumulative

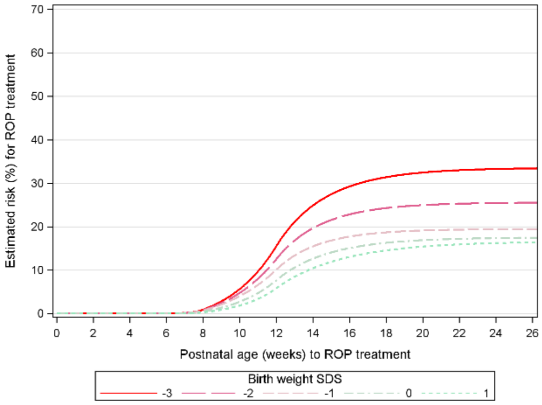

eFigure 3. Incidence of ROP Treatment by Gestational Age, Sex, and Birth Weight$_{SDS}$[3] (BWSDS)

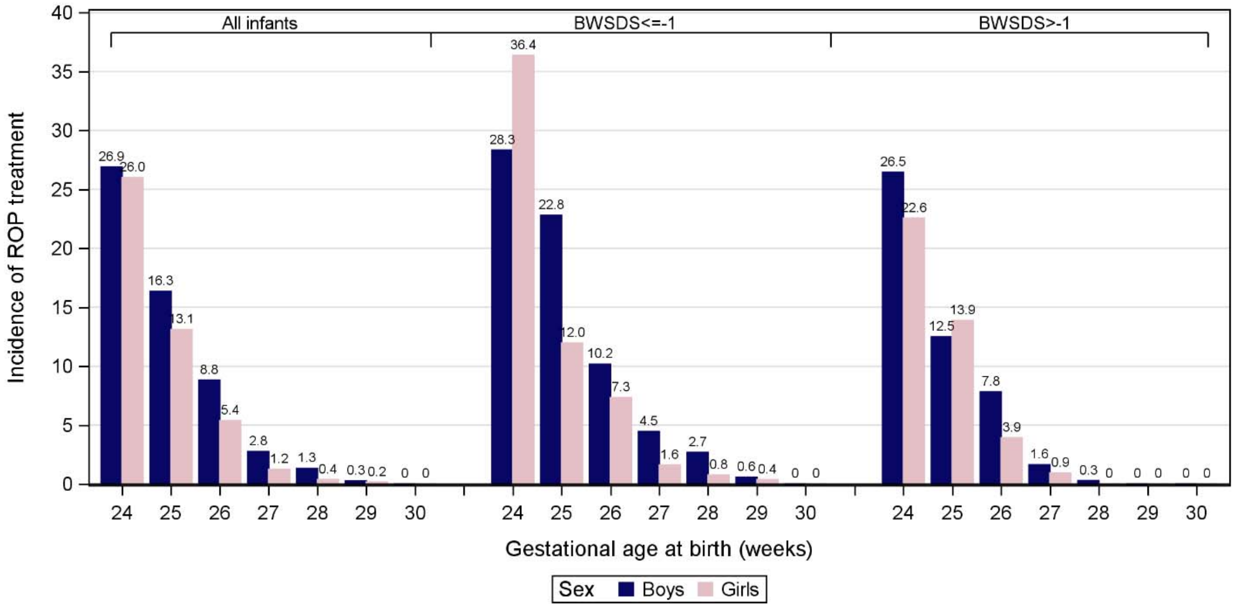

eFigure 4. Estimated Risk (%) for ROP Treatment by Gestational Age and Sex

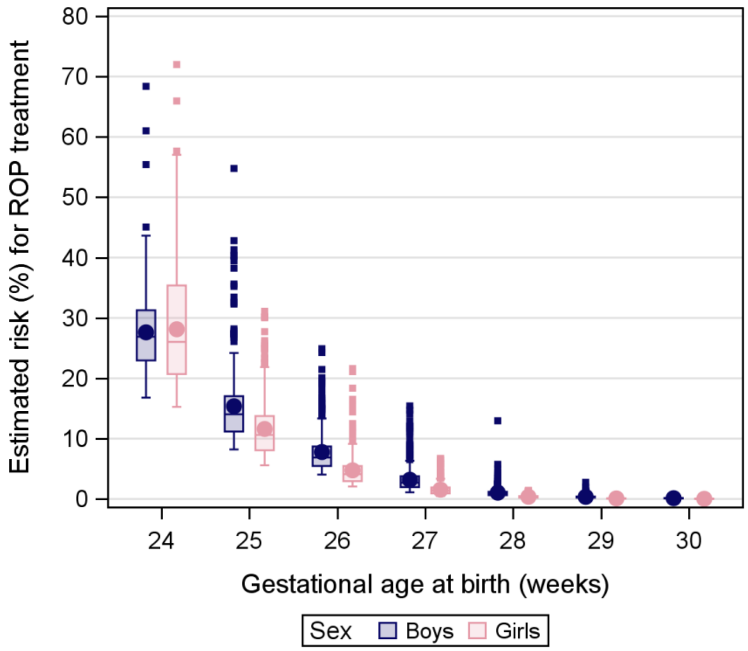

eFigure 5. ROC Curves Obtained Based on Cumulative Individual Risk for ROP Treatment for Gestational Age at Birth ≥24 Weeks From Model Group, Validation Temporal Group, Validation US Group and Validation European Group (A), by Calendar Periods in the Model Group (B), and by Race/Ethnicity in the Validation US Group (C)

A

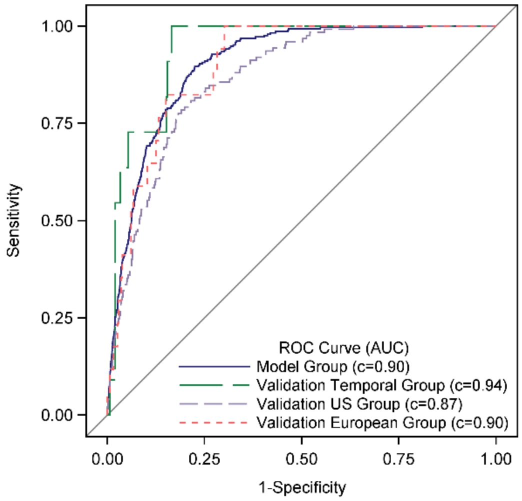

B

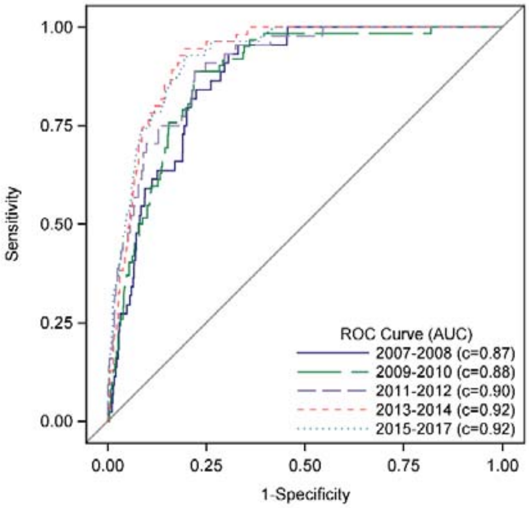

C

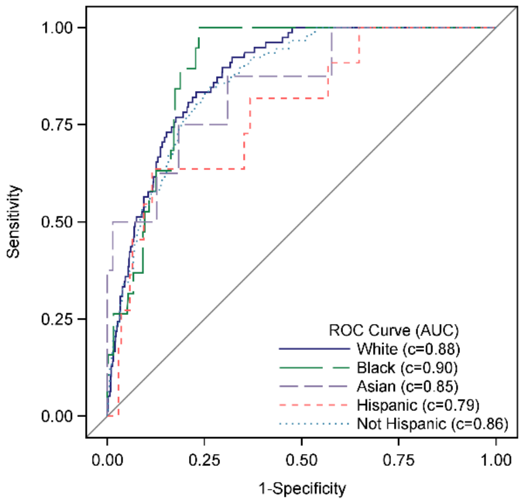

eFigure 6. Calibration Plot for Observed Proportion of ROP Treatment vs Estimated Probability Obtained From the Final Prediction Model for Gestational Age at Birth ≥24 Weeks (A), and From Cross-Validation Model for Gestational Age ≥24 Weeks (B)

A

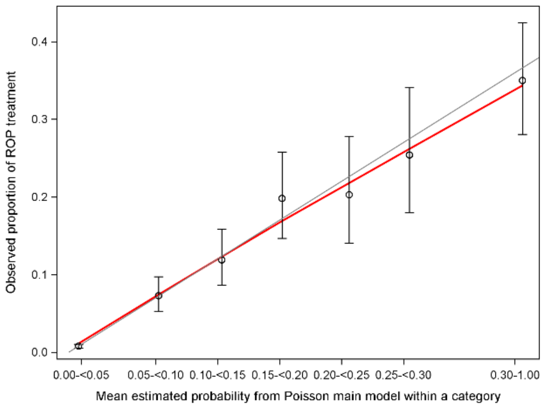

B

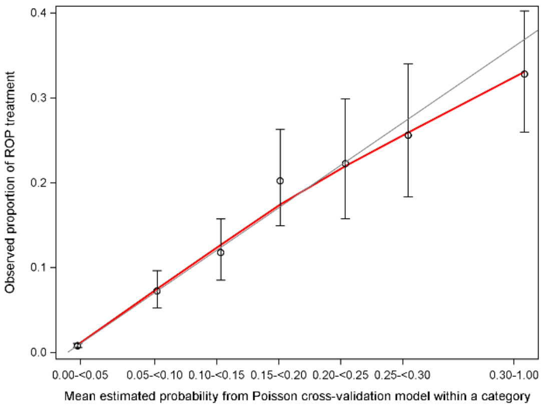

eFigure 7. ROC Curves Obtained Based on Cumulative Individual Risk for ROP Treatment for Gestational Age at Birth ≥24 Weeks From Validation US Group. DIGIROP-Birth vs CHOP-ROP (A), DIGIROP-Birth vs OMA-ROP (B), DIGIROP-Birth vs WINROP (C), and DIGIROP-Birth vs CO-ROP (D)

A

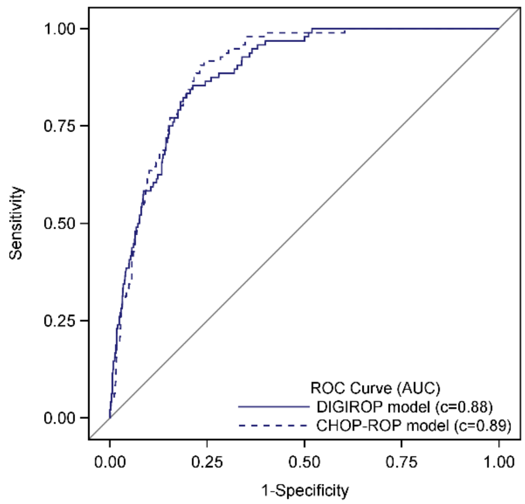

B

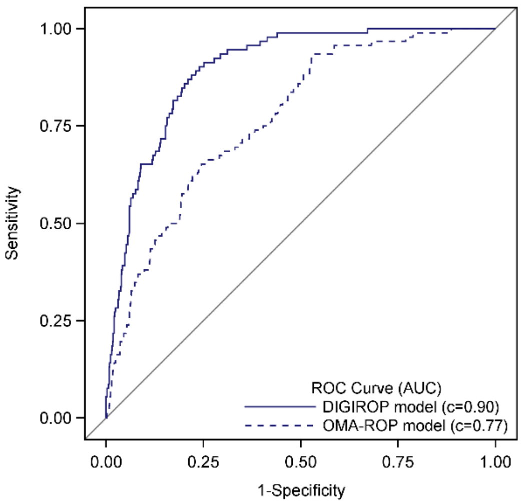

C

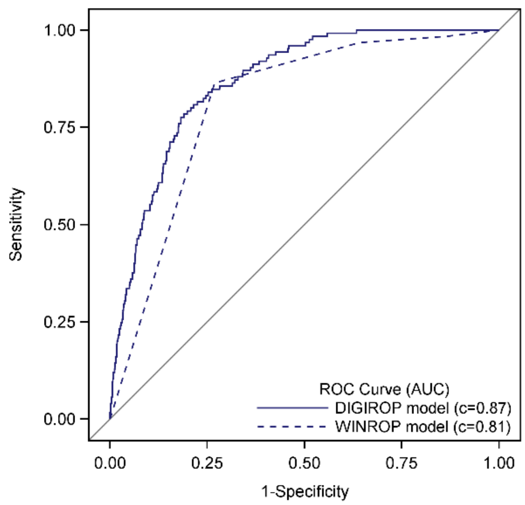

D

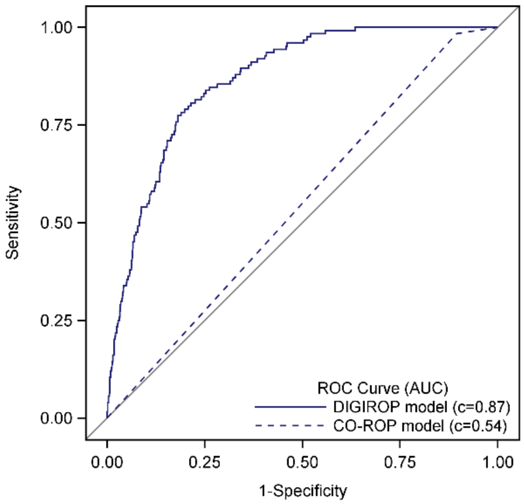

eFigure 8. Scatterplots for Individual Risk Predictions for ROP Treatment Performed on Validation US Group, Obtained From DIGIROP-Birth and CHOP-ROP Models (A), DIGIROP-Birth and CHOP-ROP Models Zoom-In Figure for Probabilities 0.0-0.1 (B), DIGIROP-Birth and OMA-ROP Models (C), and DIGIROP-Birth and WINROP Models (D). The Solid Vertical and Horizontal Gray Lines Correspond To Cut-Off Values Presented in eTable 6.

A

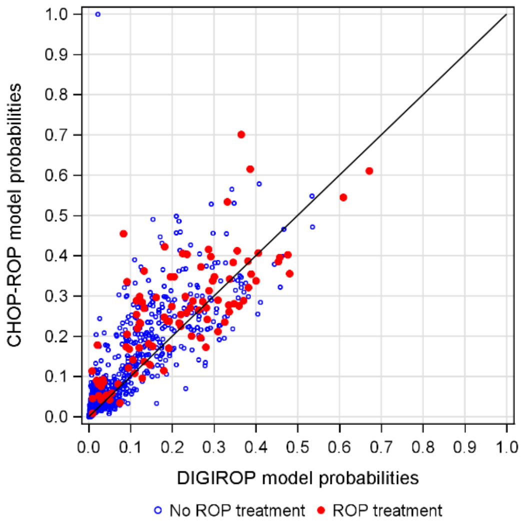

B

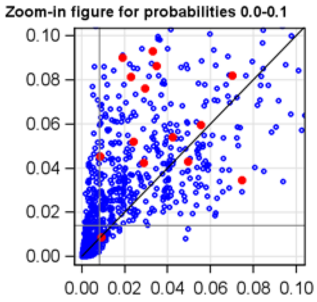

C

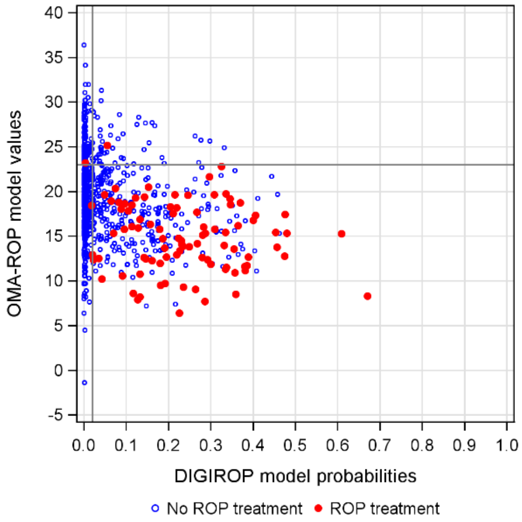

D

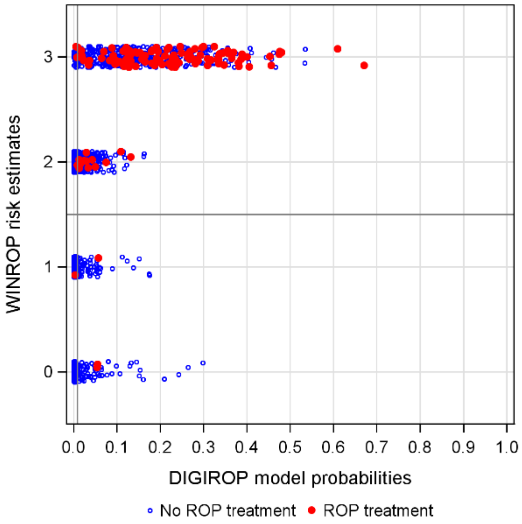

eFigure 9. Cumulative Individual Risk With 95% CI for ROP Treatment for Gestational Age at Birth <24 Weeks

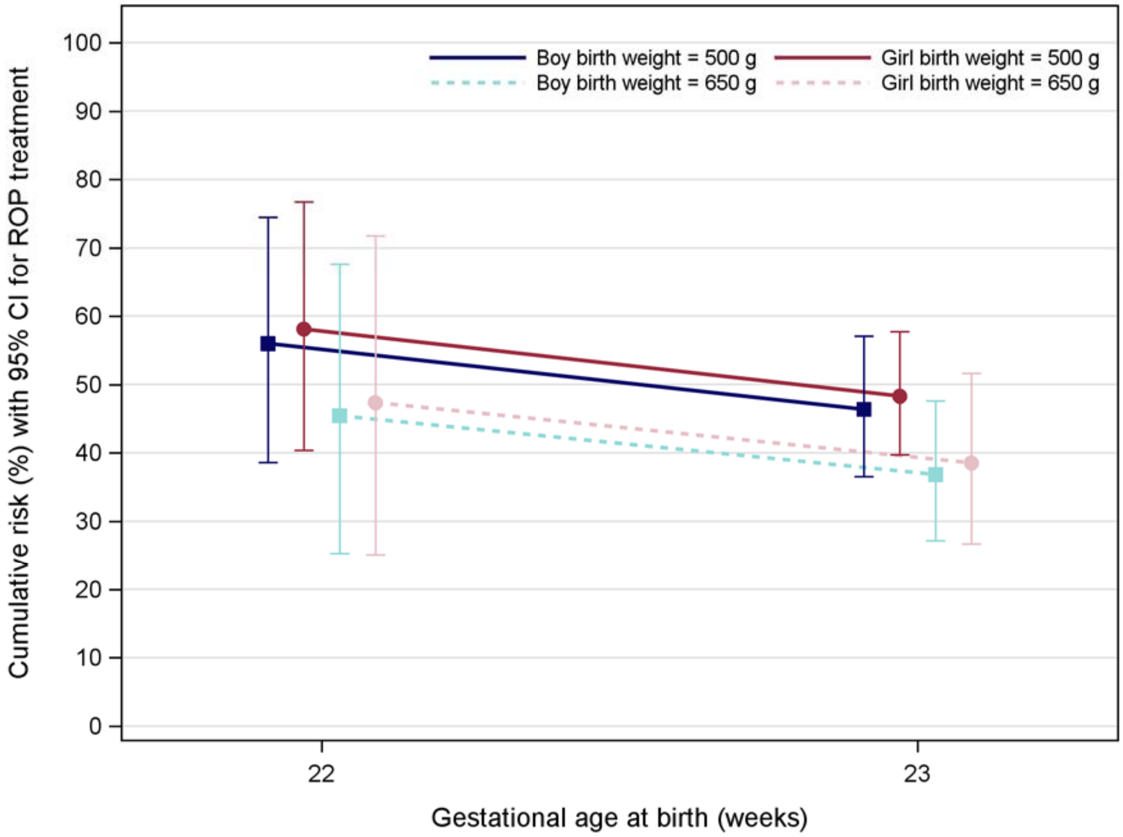

eFigure 10. ROC Curves Obtained Based on Cumulative Individual Risk for ROP Treatment for Gestational Age at Birth <24 Weeks From the Main Study, Cross-Validation, and External Population Database (A) and by Calendar Periods for the Main Study Database (B)

A **B**

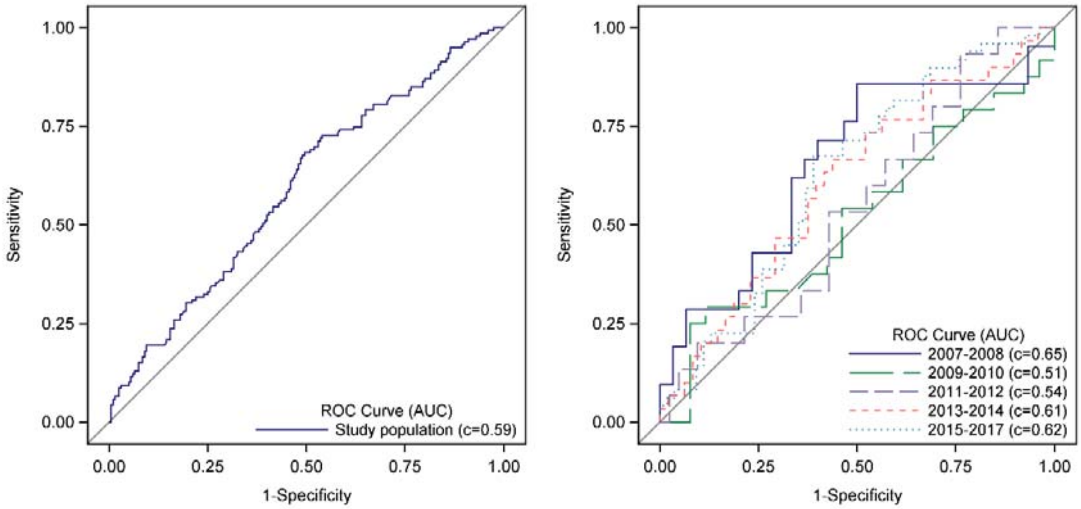

eFigure 11. Calibration Plot for Observed Proportion of ROP Treatment vs Estimated Probability Obtained From the Final Prediction Model for Gestational Age at Birth <24 Weeks (A), and From the Cross-Validation Model for Gestational Age <24 Weeks (B)

A

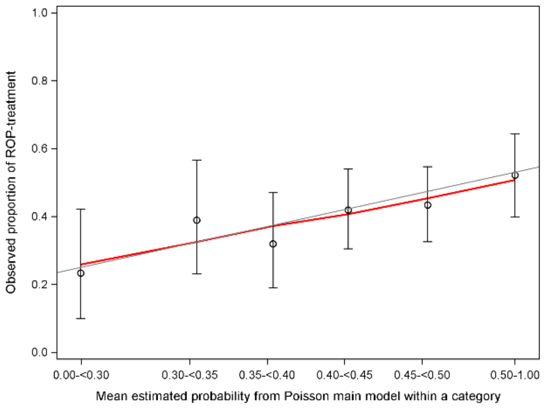

B

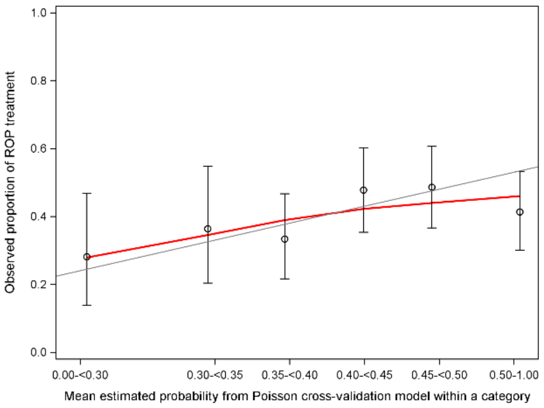

eTable 1. Birth Characteristics for Total SWEDROP Cohort by Study Population and by Maximum ROP Stage

Variable		By Study Population			By Maximum ROP Stage					
	**Total (N=7609)**	**Model Group (n=7286)**	**Validation Group (n=323)**	**P**	**No ROP (n=5182)**	**Max stage 1 (n=729)**	**Max stage 2 not treated (n=868)**	**Max stage 3 not treated (n=388)**	**Treated ROP (n=442)**	**P**
**Sex**				0.18						0.75
**Boys**	4155 (54.6%)	3991 (54.8%)	164 (50.8%)		2853 (55.1%)	359 (49.2%)	484 (55.8%)	199 (51.3%)	260 (58.8%)	
**Girls**	3454 (45.4%)	3295 (45.2%)	159 (49.2%)		2329 (44.9%)	370 (50.8%)	384 (44.2%)	189 (48.7%)	182 (41.2%)	
**Gestational Age at Birth (weeks)**	28.1 (2.1) 28.6 (21.9; 30.9) N=7609	28.0 (2.1) 28.4 (21.9; 30.9) n=7286	28.3 (2.1) 28.9 (22.6; 30.9) n=323	0.049	28.9 (1.6) 29.3 (22.3; 30.9) n=5182	27.3 (1.9) 27.4 (22.4; 30.9) n=729	26.4 (1.8) 26.3 (22.1; 30.9) n=868	25.8 (1.8) 25.6 (22.1; 30.9) n=388	24.7 (1.5) 24.6 (21.9; 29.7) n=442	<0.001
**Gestational Age at Birth (weeks)**				0.08						<0.001
**<24 Weeks**	354 (4.7%)	339 (4.7%)	15 (4.6%)		39 (0.8%)	31 (4.3%)	78 (9.0%)	64 (16.5%)	142 (32.1%)	
**24 Weeks to <28 Weeks**	2806 (36.9%)	2705 (37.1%)	101 (31.3%)		1234 (23.8%)	401 (55.0%)	608 (70.0%)	276 (71.1%)	287 (64.9%)	
**≥28 Weeks**	4449 (58.5%)	4242 (58.2%)	207 (64.1%)		3909 (75.4%)	297 (40.7%)	182 (21.0%)	48 (12.4%)	13 (2.9%)	
**Birth Weight (g)**	1119 (353) 1110 (307; 3245) n=7609	1118 (353) 1108 (307; 3245) n=7286	1142 (352) 1145 (390; 2300) n=323	0.19	1244 (318) 1247 (340; 3245) n=5182	972 (281) 940 (382; 1930) n=729	863 (249) 830 (307; 2220) n=868	794 (221) 760 (387; 1606) n=388	687 (174) 658 (370; 1700) n=442	<0.001
**Birth Weight SDS** [a,6]	-1.04 (1.38) -0.77 (-8.56; 4.93) n=7255	-1.04 (1.37) -0.77 (-8.56; 4.93) n=6947	-1.09 (1.51) -0.77 (-6.93; 2.16) n=308	0.95	-0.971 (1.342) -0.721 (-8.557; 4.925) n=5143	-1.26 (1.42) -0.95 (-7.68; 3.12) n=698	-1.22 (1.48) -0.92 (-7.10; 4.09) n=790	-1.13 (1.35) -0.84 (-6.29; 1.21) n=324	-1.13 (1.48) -0.75 (-8.06; 2.84) n=300	<0.001
**Birth Weight <-2 SDS** [a,6]				0.94						<0.001
**≥-2SDS**	5745 (79.2%)	5500 (79.2%)	245 (79.5%)		4154 (80.8%)	515 (73.8%)	584 (73.9%)	256 (79.0%)	236 (78.7%)	
**<-2SDS**	1510 (20.8%)	1447 (20.8%)	63 (20.5%)		989 (19.2%)	183 (26.2%)	206 (26.1%)	68 (21.0%)	64 (21.3%)	
**Missing**	354	339	15		39	31	78	64	142	

For categorical variables n (%) is presented.
For continuous variables mean (SD) / median (min; max) / n= is presented.
For comparison between groups the Mantel–Haenszel Chi square test was used for ordered categorical variables and the Jonckheere–Terpstra test was used for continuous variables.
[a] Birth weight SDS is available only for gestational age ≥24 weeks +0 days
[6] Reference Niklasson & Albertsson-Wikland 2002

© 2019 American Medical Association. All Rights Reserved.

eTable 2. Birth Characteristics for Validation US Group and Validation European Group

Variable	Validation US Group (n=1535)	Validation European Group (n=354)
**Sex**		
**Boys**	809 (52.7%)	185 (52.3%)
**Girls**	726 (47.3%)	169 (47.7%)
**Gestational age at birth (weeks)**	27.6 (2.1)<br>27.9 (22.7; 30.9)<br>n=1535	27.7 (2.1)<br>28.1 (22.6; 30.9)<br>n=354
**Gestational age at birth (weeks)**		
**<24 weeks**	50 (3.3%)	25 (7.1%)
**24-<28 weeks**	732 (47.7%)	128 (36.2%)
**>=28 weeks**	753 (49.1%)	201 (56.8%)
**Birth weight (g)**	1012 (322)<br>980 (378; 2240)<br>n=1535	996 (325)<br>990 (335; 2450)<br>n=354
**Birth weight SDS** [a,6]	-1.30 (1.43)<br>-1.05 (-9.95; 2.43)<br>n=1485	-1.56 (1.56)<br>-1.23 (-9.92; 2.75)<br>n=329
**Birth weight SDS** [a,6]		
**>=-2SDS**	1132 (76.2%)	225 (68.4%)
**<-2SDS**	353 (23.8%)	104 (31.6%)
**Missing**	50	25
**Birth year**		
**2005-2006**	229 (14.9%)	0 (0.0%)
**2007-2008**	901 (58.7%)	0 (0.0%)
**2009-2010**	405 (26.4%)	0 (0.0%)
**2011-2012**	0 (0.0%)	65 (18.4%)
**2013-2014**	0 (0.0%)	118 (33.3%)
**2015-2016**	0 (0.0%)	139 (39.3%)
**2017-2018**	0 (0.0%)	32 (9.0%)
**White**	820 (53.4%)	
**Black**	414 (27.0%)	
**Asian**	83 (5.4%)	
**Hispanic**	153 (10.0%)	
**Not hispanic**	899 (58.6%)	

For categorical variables n (%) is presented.
For continuous variables Mean (SD) / Median (Min; Max) / n= is presented.
[a] Birth weight SDS is available only for gestational age ≥24 weeks +0 days
[6] Reference Niklasson & Albertsson-Wikland 2002

© 2019 American Medical Association. All Rights Reserved.

eTable 3. Number, Percentage, and Follow-up Weeks for ROP Treatment, by Sex, Gestational Age at Birth, and Birth Weight$_{SDS}$

Subgroup	ROP Treatment n (%)	Follow-up Weeks Median (IQR)
**All Infants**	442 (5.8%)	21.1 (19.9–23.0)
**Boys**	260 (6.3%)	21.1 (19.9–22.9)
**Girls**	182 (5.3%)	21.3 (19.9–23.0)
**Gestational Age <24 Weeks**	142 (40.1%)	26.2 (12.9–26.7)
**Gestational Age 24-<28 Weeks**	287 (10.2%)	23.3 (22.6–24.3)
**Gestational Age ≥28 Weeks**	13 (0.3%)	20.3 (19.7–21.1)
**Birth Weight <-2 SDS** [a,6]	64 (4.2%)	21.0 (19.9–22.4)
**Birth Weight ≥-2 SDS** [a,6]	236 (4.1%)	21.1 (19.9–23.0)

Event rates per 1000 person-weeks are obtained from generalized linear models with Poisson distribution and log-link function.

[a] Birth weight SDS is available only for gestational age ≥24 weeks +0 days

[6] Reference Niklasson & Albertsson-Wikland 2002

© 2019 American Medical Association. All Rights Reserved.

eTable 4. Prediction Models for ROP Treatment for Total SWEDROP Cohort Using Only Postnatal Age and Gestational Age—Poisson Regression for Time-Varying Data

Model	Predictor	Estimate (SE)	Hazard Ratio (95% CI)	P
**Model 1**	**Intercept**	-23.7565 (4.9878)	0.00 (0.00–0.00)	<0.001
	**Postnatal Age 0–8 Weeks (by 1 week increase)**	2.2233 (0.6295)	9.24 (2.69–31.73)	<0.001
	**Postnatal Age 8–12 Weeks (by 1 week increase)**	0.4012 (0.0504)	1.49 (1.35–1.65)	<0.001
	**Postnatal Age >12 Weeks (by 1 week increase)**	-0.3430 (0.0258)	0.71 (0.67–0.75)	<0.001
**Model 2**	**Intercept**	-20.8523 (4.7984)	0.00 (0.00–0.00)	<0.001
	**Postnatal Age 0–8 Weeks (by 1 week increase)**	1.7515 (0.6061)	5.76 (1.76–18.90)	0.004
	**Postnatal Age 8–12 Weeks (by 1 week increase)**	0.4304 (0.0508)	1.54 (1.39–1.70)	<0.001
	**Postnatal Age >12 Weeks (by 1 week increase)**	-0.3536 (0.0237)	0.70 (0.67–0.74)	<0.001
	**Gestational Age 24–27 (by 1 week increase)**	-0.6372 (0.0366)	0.53 (0.49–0.57)	<0.001
	**Gestational Age >27 (by 1 week increase)**	-1.3521 (0.1579)	0.26 (0.19–0.35)	<0.001
SE = standard error; CI = confidence interval; INT = interaction term				

© 2019 American Medical Association. All Rights Reserved.

eTable 5. Estimated Probability for ROP Treatment With 95% CI for Selected Values of Birth Weight$_{SDS}$, Gestational Age at Birth, Sex, and Postnatal Age—Final Poisson Regression Model for Gestational Age at Birth ≥24 Weeks

			Cumulative Probability for ROP Treatment with 95% CI	
**Birth Weight SDS [6]**	**Gestational Age at Birth**	**Postnatal Age**	**Boys**	**Girls**
**–3**	**24 Weeks + 0 Days**	**0–8 Weeks**	0.022 (0.000–0.063)	0.023 (0.000–0.066)
		**0–12 Weeks**	0.323 (0.223–0.442)	0.340 (0.234–0.465)
		**0–20 Weeks**	0.577 (0.467–0.704)	0.600 (0.484–0.731)
	**25 Weeks + 0 Days**	**0–8 Weeks**	0.009 (0.000–0.024)	0.007 (0.000–0.020)
		**0–12 Weeks**	0.155 (0.116–0.203)	0.131 (0.097–0.172)
		**0–20 Weeks**	0.325 (0.266–0.392)	0.278 (0.226–0.340)
	**26 Weeks + 0 Days**	**0–8 Weeks**	0.004 (0.000–0.010)	0.002 (0.000–0.006)
		**0–12 Weeks**	0.070 (0.053–0.092)	0.046 (0.033–0.062)
		**0–20 Weeks**	0.164 (0.132–0.201)	0.109 (0.085–0.140)
	**27 Weeks + 0 Days**	**0–8 Weeks**	0.001 (0.000–0.004)	0.001 (0.000–0.002)
		**0–12 Weeks**	0.031 (0.020–0.044)	0.016 (0.009–0.024)
		**0–20 Weeks**	0.079 (0.057–0.105)	0.041 (0.026–0.058)
	**28 Weeks + 0 Days**	**0–8 Weeks**	0.001 (0.000–0.003)	0.000 (0.000–0.001)
		**0–12 Weeks**	0.012 (0.008–0.018)	0.005 (0.002–0.008)
		**0–20 Weeks**	0.024 (0.016–0.033)	0.009 (0.005–0.016)
	**29 Weeks + 0 Days**	**0–8 Weeks**	0.001 (0.000–0.004)	0.000 (0.000–0.001)
		**0–12 Weeks**	0.005 (0.001–0.010)	0.002 (0.000–0.004)
		**0–20 Weeks**	0.008 (0.003–0.014)	0.002 (0.000–0.006)
	**30 Weeks + 0 Days**	**0–8 Weeks**	0.000 (0.000–0.007)	0.000 (0.000–0.002)
		**0–12 Weeks**	0.002 (0.000–0.010)	0.001 (0.000–0.003)
		**0–20 Weeks**	0.003 (0.000–0.011)	0.001 (0.000–0.003)
**0**	**24 Weeks + 0 Days**	**0–8 Weeks**	0.010 (0.000–0.035)	0.011 (0.000–0.035)
		**0–12 Weeks**	0.168 (0.120–0.225)	0.178 (0.122–0.244)
		**0–20 Weeks**	0.334 (0.266–0.411)	0.351 (0.267–0.444)
	**25 Weeks + 0 Days**	**0–8 Weeks**	0.004 (0.000–0.014)	0.004 (0.000–0.011)
		**0–12 Weeks**	0.077 (0.059–0.097)	0.064 (0.048–0.083)
		**0–20 Weeks**	0.169 (0.141–0.199)	0.142 (0.115–0.174)
	**26 Weeks + 0 Days**	**0–8 Weeks**	0.002 (0.000–0.006)	0.001 (0.000–0.004)
		**0–12 Weeks**	0.034 (0.025–0.044)	0.022 (0.015–0.030)
		**0–20 Weeks**	0.081 (0.065–0.098)	0.053 (0.040–0.068)
	**27 Weeks + 0 Days**	**0–8 Weeks**	0.001 (0.000–0.002)	0.000 (0.000–0.001)
		**0–12 Weeks**	0.015 (0.009–0.022)	0.008 (0.004–0.012)
		**0–20 Weeks**	0.038 (0.026–0.051)	0.019 (0.012–0.028)
	**28 Weeks + 0 Days**	**0–8 Weeks**	0.000 (0.000–0.002)	0.000 (0.000–0.001)
		**0–12 Weeks**	0.006 (0.003–0.009)	0.002 (0.001–0.004)
		**0–20 Weeks**	0.011 (0.007–0.016)	0.004 (0.002–0.008)
	**29 Weeks + 0 Days**	**0–8 Weeks**	0.000 (0.000–0.003)	0.000 (0.000–0.001)
		**0–12 Weeks**	0.002 (0.000–0.006)	0.001 (0.000–0.002)
		**0–20 Weeks**	0.004 (0.001–0.008)	0.001 (0.000–0.003)
	**30 Weeks + 0 Days**	**0–8 Weeks**	0.000 (0.000–0.006)	0.000 (0.000–0.002)
		**0–12 Weeks**	0.001 (0.000–0.007)	0.000 (0.000–0.002)
		**0–20 Weeks**	0.001 (0.000–0.007)	0.000 (0.000–0.002)

© 2019 American Medical Association. All Rights Reserved.

Estimated probability is obtained from Poisson regression for time-varying data.
Confidence intervals are computed by using bootstrapping method of 1000 samples.
CI = confidence interval
[6] Reference Niklasson & Albertsson-Wikland 2002

© 2019 American Medical Association. All Rights Reserved.

eTable 6. DIGIROP-Birth vs CHOP-ROP, OMA-ROP, WINROP and CO-ROP. AUC, Sensitivity, Specificity, PPV and NPV

	**DIGIROP-Birth vs CHOP-ROP**		**DIGIROP-Birth vs CHOP-ROP**		**DIGIROP-Birth vs OMA-ROP**		**DIGIROP-Birth vs WINROP**		**DIGIROP-Birth vs CO-ROP**	
	**CHOP-ROP model [1]**	**DIGIROP-Birth model [1]**	**CHOP-ROP model [1]**	**DIGIROP-Birth model [1]**	**OMA-ROP model [2]**	**DIGIROP-Birth model [2]**	**WINROP model [3]**	**DIGIROP-Birth model [3]**	**CO-ROP model [4]**	**DIGIROP-Birth model [4]**
**Number of infants with available data**	1442	1442	1442	1442	863	863	1485	1485	1465	1465
**AUC**	0.89	0.88	0.89	0.88	0.77	0.90	0.81	0.87	0.54	0.87
**Cutoff [5]**	0.0034 probability	0.0076 probability	0.0140 probability	0.0083 probability	23 g/d	0.0200 probability	Risk=[2, 3] (Alarm)	0.0089 probability	Risk=1 (Alarm)	0.0076 probability
**Sensitivity**	96/96 (100%)	96/96 (100%)	95/96 (99.0%)	95/96 (99.0%)	90/92 (97.8%)	90/92 (97.8%)	121/125 (96.8%)	121/125 (96.8%)	122/124 (98.4%)	122/124 (98.4%)
**Specificity**	450/1346 (33.4%)	647/1346 (48.1%)	598/1346 (44.4%)	658/1346 (48.9%)	173/771 (22.4%)	448/771 (58.1%)	487/1360 (35.8%)	671/1360 (49.3%)	141/1341 (10.5%)	642/1341 (47.9%)
**PPV**	96/992 (9.7%)	96/795 (12.1%)	95/843 (11.3%)	95/783 (12.1%)	90/688 (13.1%)	90/413 (21.8%)	121/994 (12.2%)	121/810 (14.9%)	122/1322 (9.2%)	122/821 (14.9%)
**NPV**	450/450 (100%)	647/647 (100%)	598/599 (99.8%)	658/659 (99.8%)	173/175 (98.9%)	448/450 (99.6%)	487/491 (99.2%)	671/675 (99.4%)	141/143 (98.6%)	642/644 (99.7%)

[1] Analyses performed on Validation US Group where longitudinal weights for two latest weeks not differing more than 8 days in-between are available for 1442 infants.

[2] Analyses performed on Validation US Group where longitudinal weights, at 36 weeks or before for infants receiving ROP treatment before 36th postmenstrual week, are available for 863 infants.

[3] Analyses performed on Validation US Group where longitudinal weights were used for WINROP risk calculation as published in Wu et al 2012. All 1485 infants available with GA 24-30 could be included in this calculation.

[4] Analyses performed on Validation US Group where longitudinal weight at 4 weeks postnatal age was available, for 1465 infants.

[5] The cut-off for DIGIROP-Birth model was selected based on sensitivity obtained applying the published cut-offs from the CHOP-ROP, OMA-ROP, WINROP and CO-ROP models.

© 2019 American Medical Association. All Rights Reserved.

eTable 7. Final Prediction Model for ROP Treatment for Infants With Gestational Age at Birth<24 Weeks—Poisson Regression for Time-Varying Data

Predictor	Estimate (SE)	Hazard Ratio (95% CI)	P
**Intercept**	-13.7974 (2.3106)	0.00 (0.00–0.00)	<0.001
**Postnatal Age 0–12 Weeks (by 1 week increase)**	0.7570 (0.1073)	2.13 (1.73–2.63)	<0.001
**Postnatal Age >12 Weeks (by 1 week increase)**	-0.3797 (0.0414)	0.68 (0.63–0.74)	<0.001
**Gestational Age (by 1 week increase)**	-0.2760 (0.2046)	0.76 (0.51–1.13)	0.18
**Sex (1 = boys, 2 = girls)**	0.0578 (0.1742)	1.06 (0.75–1.49)	0.74
**Birth Weight ≤550 g (by 50 g increase)**	0.1459 (0.1281)	1.16 (0.90–1.49)	0.25
**Birth Weight >550 g (by 50 g increase)**	-0.2249 (0.1001)	0.80 (0.66–0.97)	0.03
SE = standard error; CI = confidence interval			

© 2019 American Medical Association. All Rights Reserved.

eTable 8. Estimated Probability for ROP Treatment With 95% CI for Selected Values of Birth Weight, Gestational Age at Birth, Sex, and Postnatal Age—Final Poisson Regression Model for Gestational Age at Birth <24 Weeks

			**Cumulative Probability for ROP Treatment with 95% CI**	
**Birth Weight (g)**	**Gestational Age at Birth**	**Postnatal Age**	**Boys**	**Girls**
**500**	**22 Weeks + 0 Days**	**0–8 Weeks**	0.014 (0.000–0.035)	0.014 (0.000–0.037)
		**0–12 Weeks**	0.247 (0.137–0.371)	0.259 (0.141–0.391)
		**0–20 Weeks**	0.560 (0.390–0.738)	0.581 (0.400–0.765)
	**23 Weeks + 0 Days**	**0–8 Weeks**	0.010 (0.000–0.025)	0.011 (0.000–0.026)
		**0–12 Weeks**	0.193 (0.135–0.261)	0.204 (0.147–0.268)
		**0–20 Weeks**	0.464 (0.371–0.566)	0.483 (0.395–0.578)
**650**	**22 Weeks + 0 Days**	**0–8 Weeks**	0.010 (0.000–0.028)	0.011 (0.000–0.030)
		**0–12 Weeks**	0.189 (0.076–0.320)	0.199 (0.073–0.344)
		**0–20 Weeks**	0.454 (0.250–0.675)	0.474 (0.247–0.714)
	**23 Weeks + 0 Days**	**0–8 Weeks**	0.008 (0.000–0.019)	0.008 (0.000–0.020)
		**0–12 Weeks**	0.147 (0.093–0.208)	0.155 (0.092–0.225)
		**0–20 Weeks**	0.368 (0.268–0.479)	0.385 (0.265–0.515)

Estimated probability is obtained from Poisson regression for time-varying data.
Confidence intervals are computed by using bootstrapping method of 1000 samples.
CI = confidence interval

© 2019 American Medical Association. All Rights Reserved.

eReferences

1. Hosmer DW, Lemeshow S. Applied logistic regression. 2nd Edition, John Wiley & Sons, Inc., New York. 2000 doi:10.1002/0471722146.
2. Binenbaum G, Ying G, Tomlinson LA. Validation of the Children's Hospital of Philadelphia Retinopathy of Prematurity (CHOP ROP) Model. JAMA Ophthalmol. 2017;135:871-877.
3. McCauley K, Chundu A, Song H, High R, Suh D. Implementation of a Clinical Prediction Model Using Daily Postnatal Weight Gain, Birth Weight, and Gestational Age at Risk Stratify ROP. J Pediatr Ophthalmol Strabismus. 2018;55:326-334.
4. Lofqvist C, Hansen-Pupp I, Andersson E, et al. Validation of a new retinopathy of prematurity screening method monitoring longitudinal postnatal weight and insulinlike growth factor I. Arch Ophthalmol 2009;127:622-7.
5. Wu C, Löfqvist C, Smith LEH, VanderVeen DK, Hellström A. Importance of Early Postnatal Weight Gain for Normal Retinal Angiogenesis in Very Preterm Infants: A Multicenter Study Analyzing Weight Velocity Deviations for the Prediction of Retinopathy of Prematurity. Arch Ophthalmol. 2012; 130:992-999.
6. Niklasson A, Albertsson-Wikland K. Continuous growth reference from 24th week of gestation to 24 months by gender. BMC Pediatr 2008;8:8.

© 2019 American Medical Association. All Rights Reserved.
